# Natural Product-Based Upconversion–Downshifting Photosensitizers in Photodynamic Therapy

**DOI:** 10.3390/ph19071062

**Published:** 2026-07-09

**Authors:** Xiaohui Li, Siu Kan Law, Albert Wing Nang Leung, Mingfang Li, Chuanshan Xu

**Affiliations:** 1 Guangdong Provincial Key Laboratory of Major Obstetric Diseases, Department of Dermatology, Guangdong Provincial Clinical Research Center for Obstetrics and Gynecology, The Third Affiliated Hospital, Guangzhou Medical University, Guangzhou 510150, China; 15812438892@163.com (X.L.); mingfangli@gzhmu.edu.cn (M.L.); 2 Independent Researcher, Hong Kong SAR, China; siukanlaw@hotmail.com; 3 School of Graduate Studies, Lingnan University, Tuen Mun, Hong Kong SAR, China; albertleung@ln.edu.hk; 4 Guangzhou Municipal and Guangdong Provincial Key Laboratory of Molecular Target & Clinical Pharmacology, The NMPA and State Key Laboratory of Respiratory Disease, School of Pharmaceutical Sciences, Guangzhou Medical University, Guangzhou 511436, China

**Keywords:** lanthanide, natural product, photodynamic therapy, upconversion nanoparticles (UCNPs), upconversion–downshifting nanoparticles (UDNPs)

## Abstract

Natural product-based upconversion photosensitizers (PSs) have emerged as innovative agents in photodynamic therapy (PDT). Lanthanide ions such as Yb^3+^, Er^3+^, Nd^3+^, Gd^3+^, and Tm^3+^ have unique photophysical properties and biocompatibility, exhibiting sharp 4f–4f transitions and long-lived excited states involving the dual luminescence processes, upconversion and downshifting. Natural product photosensitizers (PSs), including coumarin, riboflavin, curcumin, chlorophyll derivatives, and hypocrellin, offer superior safety profiles compared with synthetic PSs. Recent advances in upconversion nanoparticles (UCNPs) and upconversion–downshifting nanoparticles (UDNPs) for the generation of ROS in PDT have been evaluated. This narrative review surveyed the literature published between 1995 and 2026 across multiple electronic databases, including WanFang Data, PubMed, ScienceDirect, Scopus, Web of Science, Springer Link, SciFinder, and the China National Knowledge Infrastructure (CNKI), without language restrictions. The search focused on studies related to photodynamic therapy, lanthanide photophysics, and natural product photosensitizers such as coumarin, riboflavin, curcumin, chlorophyll derivatives, and hypocrellin, as well as nanoplatforms involving upconversion (UCNPs) and upconversion–downshifting nanoparticles (UDNPs). Relevant publications were identified and synthesized to integrate advances in lanthanide photophysics, natural product PSs, and nanoplatform design into a conceptual framework. Natural product-based upconversion PSs for PDT have the advantages of low dark toxicity, biocompatibility, and multimodal actions. Lanthanide-enhanced systems overcome these issues, including shallow tissue penetration, photobleaching, and relatively low singlet oxygen quantum yields. Thus, natural product-based upconversion PSs in PDT are an innovative strategy, but bridging preclinical promise with clinical translation remains a critical future challenge.

## 1. Introduction

**Figure 1:**
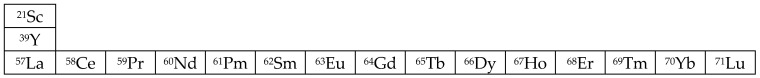
Lanthanides in rare-earth elements (REEs).

Lanthanides belong to a group of rare-earth elements (REEs), which have 15 metallic elements at the atomic numbers 57 to 71, from La to Lu together with yttrium (Y) and scandium (Sc), making a total of 17 elements (Figure 1) [1]. They have partially filled 4f subshells, and the electrons within these orbitals give rise to characteristic, narrow, line-like emissions with unique photophysical properties [2]. The lanthanide ions exhibit higher Lewis acidity and possess distinct coordination chemistry, enabling selective uptake, intracellular trafficking, and incorporation into enzymatic systems to facilitate and enhance the photophysical performance for clinical applications [3]. For example, lanthanide compounds are widely applied as diagnostic and prognostic probes in clinical practice and have also been explored as anticancer agents. The lanthanide complexes serve as effective X-ray contrast materials, and lanthanide chelate-based agents are extensively employed as contrast enhancers in magnetic resonance imaging (MRI) for radiological analysis [4].

Currently, lanthanide-based small molecules and nanomaterials have been investigated in photodynamic therapy (PDT) because of their unique photophysical properties [5]. Typically, PDT is induced by ultraviolet–visible (UV-vis) and X-ray light. The UV-vis PDT represents a promising approach for cancer treatment, since it has the function of tumor targeting. A photosensitizer (PS) is initially excited to a singlet state upon the UV-vis irradiation, then passed through the intersystem crossing (ISC) to form a triplet state. This triplet state reacts with molecular oxygen to generate singlet oxygen (^1^O_2_) for destroying malignant cells [6]. The lanthanide-based compounds used in PDT facilitate intersystem crossing (ISC) through the heavy-atom effect, thereby promoting the efficient generation of ^1^O_2_ [7]. Similarly, X-ray PDT is excited optical luminescence from lanthanide-based nanoparticles, which offers an attractive alternative to conventional light sources, owing to its potential for deep tissue penetration [8].

**Figure 2:**
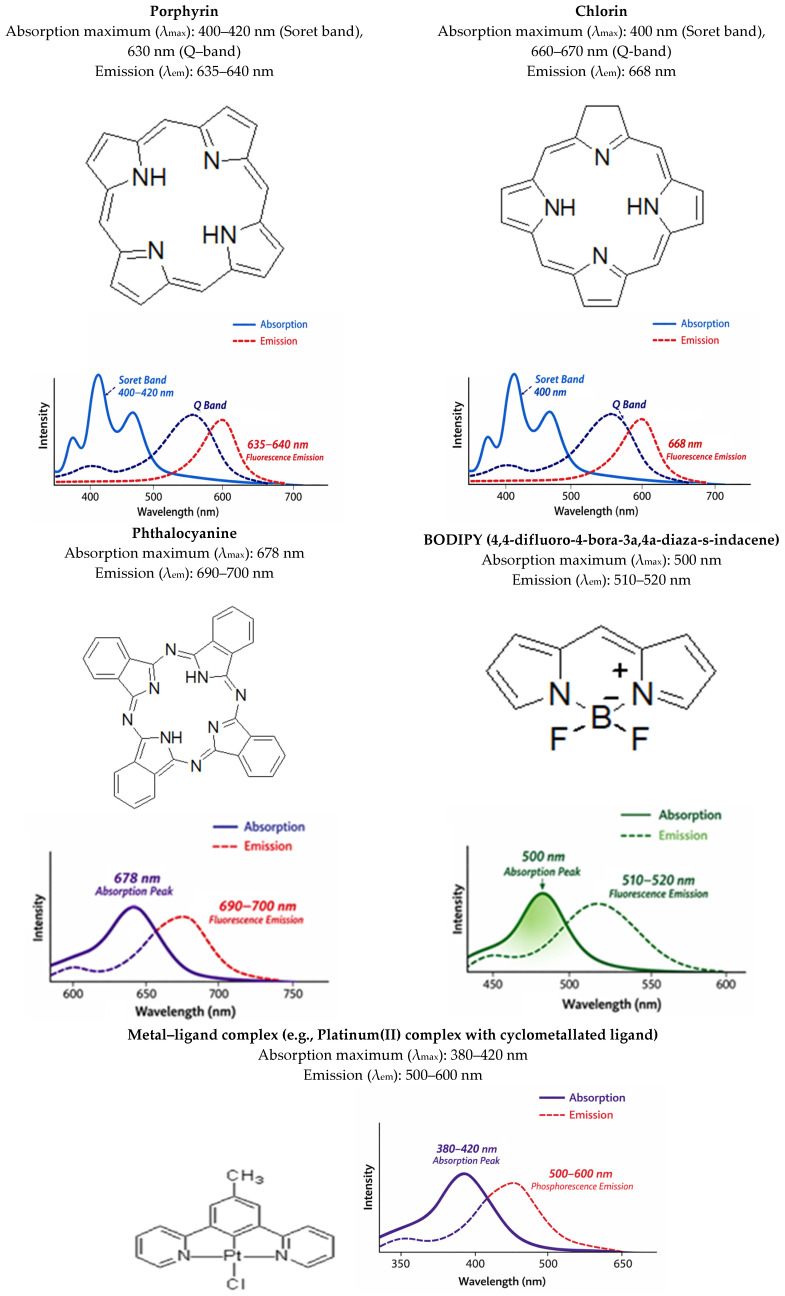
Chemical structures of chemically synthesized PSs.

**Figure 3:**
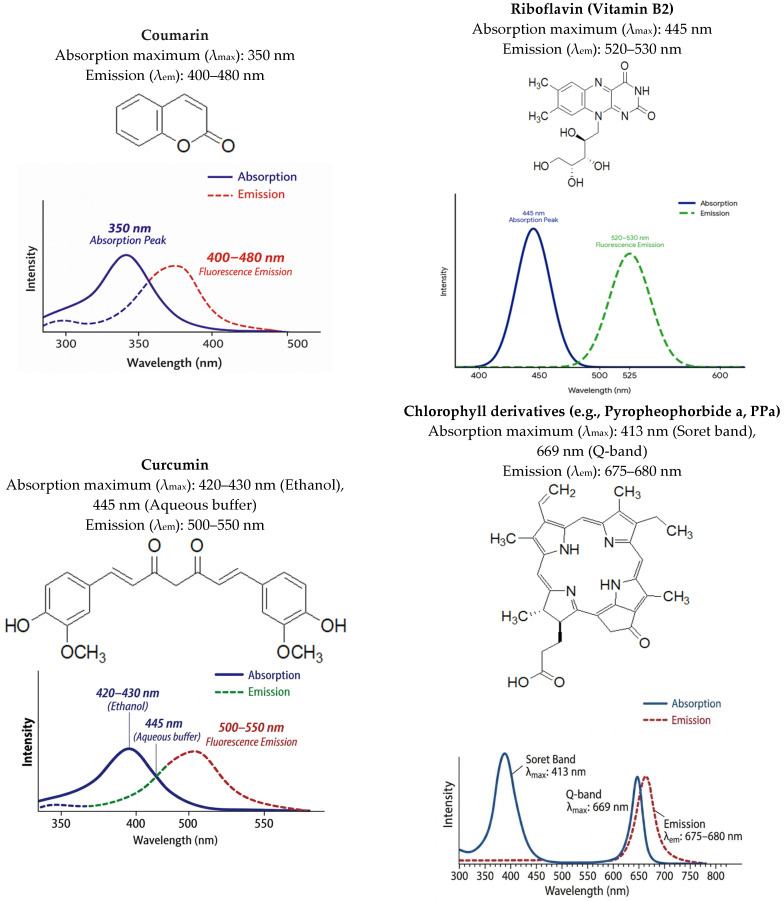

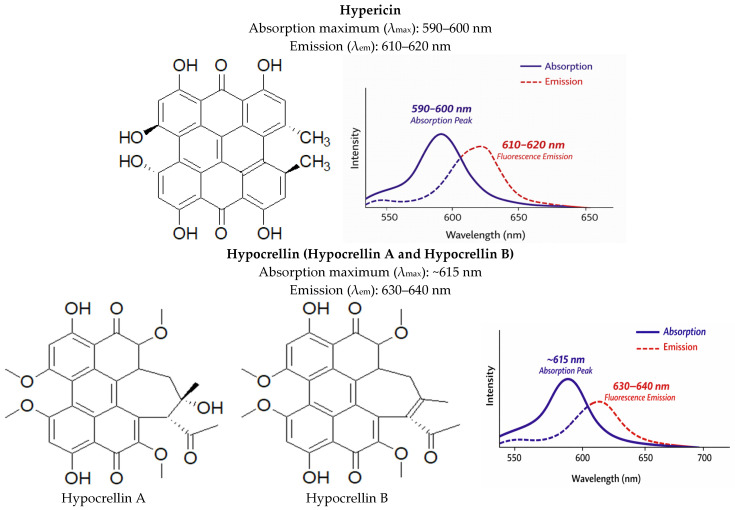
Chemical structures of natural product PSs.

PS is an important component in PDT. There are two major types of PSs, including chemically synthesized agents and natural products. Chemically synthesized PSs like porphyrins [9] and chlorins [10] occur naturally but are widely synthesized for PDT, phthalocyanines [11], BODIPY dyes that are entirely synthetic [12], and metal–ligand complexes [13] (Figure 2), whilst natural product PSs include coumarin [14], riboflavin [15], curcumin [16], chlorophyll derivatives (e.g., pyropheophorbide a, PPa) [17], hypericin, and hypocrellins [18] (Figure 3). Natural product PSs have a higher biocompatibility, minimal systemic toxicity, and a lower risk of long-term side effects compared with chemically synthesized PSs [19]. However, they usually have poor aqueous solubility, low bioavailability, instability, and sometimes short wavelengths, resulting in inconsistent therapeutic effect on clinical studies [20]. Thus, there are several approaches to overcoming these issues, such as natural product-based upconversion PSs in PDT.

Compared with the prior review on natural product PSs in PDT and other recent studies, the present article provides a distinct perspective by focusing on the integration of natural product PSs with lanthanide-based upconversion and upconversion–downshifting nanoplatforms. This UCNP/UDNP-plus-natural-product framework leads the rare-earth photophysics to overcome the limitations of natural PSs, such as poor solubility, short excitation wavelengths, and low bioavailability, while preserving their biocompatibility and multimodal actions. Specifically, this aims to discuss the role of lanthanide elements, focusing on the principles and recent advances in their integration with upconversion nanoplatforms combined with natural products as PSs, rather than lanthanide-based PDT in general. Furthermore, the PDT principle arising from the unique photophysical properties of natural product-based upconversion PSs is emphasized, as well as their research progress and clinical studies in future aspects. Thus, the value lies in synthesizing advances in lanthanide photophysics, natural product chemistry, and nanotechnology into a unified narrative, offering a roadmap for future translational PDT strategies.

## 2. Lanthanide Ions

Ytterbium (Yb^3+^), Erbium (Er^3+^), Neodymium (Nd^3+^), and Gadolinium (Gd^3+^) are lanthanide ions frequently employed in PDT, as the upconversion and downconversion luminescence processes applied overcome limitations such as poor tissue penetration by visible light [21]. Lanthanide ions possess multiple 4f excitation levels, while the completely filled 5s^2^ and 5p^6^ subshells shield the 4f electrons. This shielding produces sharp 4f–4f transition bands and long-lived excited states, enabling sequential photon absorption.

**Figure 4:**
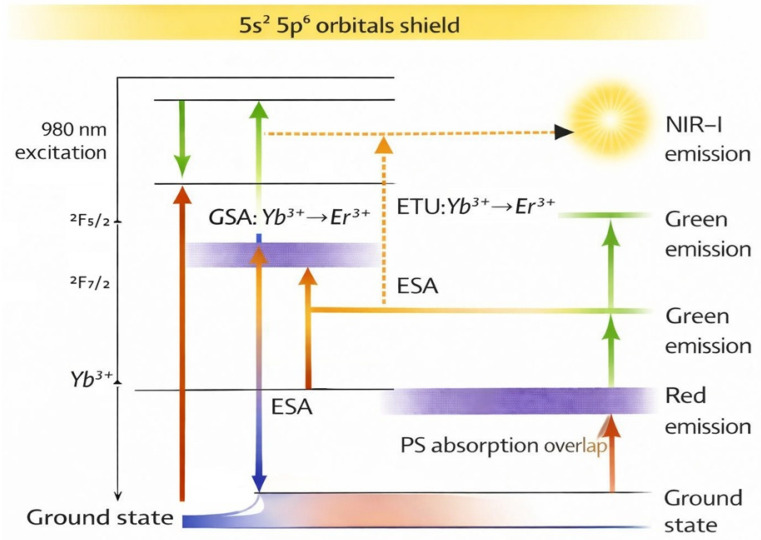
Partial energy-level diagram of the Yb^3+^–Er^3+^ upconversion system under 980 nm excitation. Upward arrows represent excitation processes, including ground-state absorption (GSA, blue/orange) and excited-state absorption (ESA, orange). Downward arrows denote radiative transitions yielding NIR-I (yellow dashed), green, and red emissions. Dashed orange arrows indicate energy transfer upconversion (ETU) pathways from Yb^3+^ to Er^3+^. Color shaded bands highlight key functional states, including the spectral overlap region for PS absorption.

Yb^3+^ ions absorb near-infrared photons (^2^F_7_/_2_→^2^F_5_/_2_) and transfer energy to Er^3+^ ions via energy-transfer upconversion (ETU: Yb^3+^→Er^3+^ ^4^I_11_/_2_). Er^3+^ ions undergo ground-state absorption (GSA: ^4^I_15_/_2_→^4^I_11_/_2_) and excited-state absorption (ESA: ^4^I_11_/_2_→^2^H_11_/_2_/^4^S_3_/_2_), leading to characteristic emissions. The radiative transitions include NIR-I emission (^4^I_11_/_2_→^4^I_15_/_2_), green emission (^2^H_11_/_2_/^4^S_3_/_2_→^4^I_15_/_2_), and red emission (^4^F_9_/_2_→^4^I_15_/_2_). Notably, the Er^3+^ red emission overlaps with the photosensitizer absorption band, enabling photodynamic activation [22]. The shielding effect of the 5s^2^5p^6^ orbitals is indicated in Figure 4.

These photophysical characteristics enhance light harvesting and mitigate the problems of short excitation wavelengths and limited tissue penetration [23]. Therefore, upconversion processes are particularly relevant, as they enable natural product PSs to be activated by NIR light for deeper tissue PDT. Trivalent lanthanides (Ln^3+^) like Yb^3+^, Er^3+^, Nd^3+^, and Gd^3+^ are most frequently employed in PDT because their higher Lewis acidity and strong spin–orbit coupling facilitate intersystem crossing (ISC), thereby enhancing singlet oxygen generation [21,23]. In contrast, lower oxidation states (e.g., Ln^2+^) are less stable in biological environments and often reduce photophysical efficiency, leading to diminished ROS yields. Thus, there is a trend for the stable trivalent lanthanides to consistently support efficient energy transfer to natural product PSs and enable reliable ROS generation, while divalent ions (lower oxidation states) are rarely used in PDT applications [24,25].

### 2.1. Upconversion

**Figure 5:**
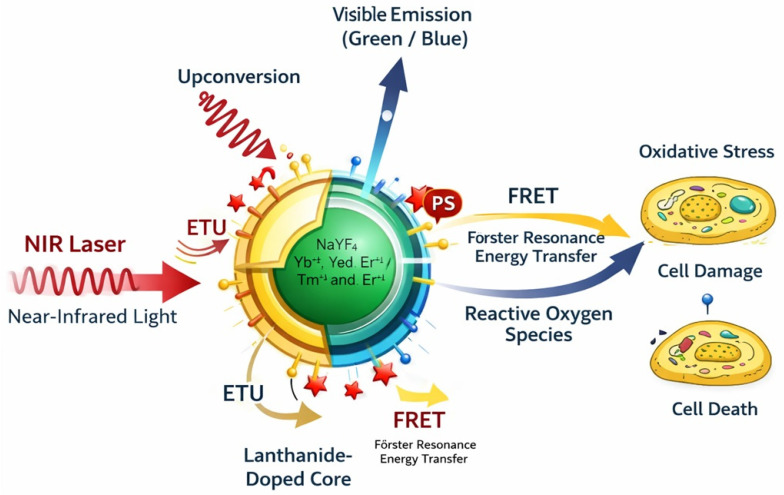
Schematic of photodynamic therapy using lanthanide-doped nanoparticles. Excitation and Energy Transfer (Left): The thick red arrow represents the NIR laser excitation (Near-Infrared Light). The wavy red arrows indicate upconversion luminescence, and the curved brown/maroon loops denote energy transfer upconversion (ETU) pathways within the lanthanide-doped core NaYF_4_. Emission and PDT activation center: The straight upward blue arrow represents visible emission (green/blue light). The curved gradient arrows (yellow/blue) signify Förster Resonance Energy Transfer (FRET) from the nanoparticle core to the surface-anchored PS, represented by the red stars. Biological Response (Right): The generation of ROS triggers intracellular pathways leading from oxidative stress and cell damage to ultimate cell death.

This is an optical process in which lower-energy photons are absorbed and converted into higher-energy emission, typically achieved using high-quality nanoparticles doped with rare-earth (lanthanide) ions [24], called upconversion nanoparticles (UCNPs). These allow deeper-penetrating near-infrared light at 980 nm, or 810 nm for PDT. For example, NaYF_4_ nanoparticles doped with Yb^3+^ and Er^3+^ or Tm^3+^ and Er^3+^, which are covalently conjugated to PS or directly adsorbed onto the coating or shell. This configuration enables Förster resonance energy transfer (FRET) to the PS, thereby transducing near-infrared (NIR) light into reactive oxygen species (ROS) to induce cell death [25]. For example, Yb^3+^ absorbs NIR light and transfers energy to Er^3+^ via energy transfer upconversion (ETU), leading to visible green and red emissions. The red emission overlaps with the photosensitizer absorption band, enabling FRET to the PS. The excited PS generates ROS, which induce oxidative stress, cell damage, and ultimately cell death (Figure 5).

### 2.2. Downconversion vs. Downshifting

Downconversion is an optical process known as quantum cutting, where a material absorbs a single, or high-energy photon from UV or blue light, which splits it into two lower-energy photons from near-infrared or visible light. Er^3+^-doped nanoparticles can exhibit dual optical processes: upconversion luminescence in the first near-infrared window (NIR-I, 700–950 nm), which overlaps with PS absorption and is advantageous for PDT, and downshifting luminescence in the second near-infrared window (NIR-II, 1000–1700 nm), which provides deeper tissue penetration and high-contrast imaging but does not directly contribute to ROS generation [26]. The therapeutic effect arises from the UCNP-activated PS under NIR irradiation. Thus, lanthanide nanoparticles excel at imaging deep within biological tissues as they are excited by NIR light and emit even longer NIR wavelengths. The lanthanide-based nanoparticles exploit dual luminescence mechanisms involving upconversion from NIR excitation to visible emission and downshifting from UV/visible excitation to NIR emission.

Downshifting (Stokes emission) involves absorption of one photon and emission of a single photon with a lower energy. This is the physically correct description for Er^3+^ and Ho^3+^ NIR-II emission systems, where visible or UV excitation leads to longer-wavelength near-infrared output. This improves tissue penetration and reduces scattering, making it highly relevant for bioimaging and PDT. The Er^3+^/Ho^3+^ NIR-II systems are classified as downshifting, not quantum cutting. An acronym UDNP refers to upconversion–downshifting nanoparticles. Quantum-cutting downconversion is mentioned only in the Tb^3+^–Yb^3+^ or Pr^3+^–Yb^3+^ systems.

## 3. Natural Product-Based UCNP Systems in PDT

These have two incorporated dual luminescence processes, including upconversion and downshifting as described above. Over the past three decades (1995–2026), lanthanide-based PDT has evolved from simple UCNP constructs to multifunctional nanoplatforms with enhanced luminescence and therapeutic precision.

### 3.1. Upconversion Nanoparticle (UCNP) System

In early studies, this focused on Yb^3+^/Er^3+^ in hosts such as Gd_2_O_3_ and NaYF_4_, coupled with zinc phthalocyanine, demonstrating efficient ROS generation under 980 nm excitation with singlet oxygen quantum yields around 0.50 to 0.63. Another was the Nd^3+^-sensitized UCNP, which achieved 808 nm excitation, reducing overheating and improving tissue penetration, with chlorin e6 and TiO_2_ shells acting as PSs. Functionalization strategies emerged, such as antibody conjugation, mitochondria-targeting triphenylphosphine, and dual-PS systems, such as RB, ZnPc, Ce6, and MC540, achieving Φ_Δ_ values of 0.45–0.65. A novel molecular sensitizer (FUCP-1), biomimetic UCNPs with CD73 blockade and dual-PS nanoplatforms, enhanced ROS yield and immunogenic apoptosis. There were antibacterial/antifungal PDT, or multicolor UCNPs, MOF/TiO_2_ composites, and photoswitchable systems that allowed multimodal PDT, excitation-dependent emission, and hypoxia relief, with Φ_Δ_ values up to 0.66. The integrated mesoporous polydopamine coatings for dual PDT/PTT therapy possessed an efficiency of 0.60. These progressions highlighted a shift from single PS, 980 nm systems with multifunctional, mitochondria-targeted, hypoxia-responsive, deeper tissue penetration, and dual-mode nanoplatforms for improving therapeutic efficacy and imaging guidance of PDT (Table 1).

In some cases, the coordination of metal ions to natural product PSs with ionophore-like behavior, which alters their photophysical properties. Strong coordination may disrupt the conjugated π-system of the PS or change its electronic distribution, leading to reduced absorption efficiency and diminished ROS generation. For example, metal–hypocrellin complexes have been reported to lose photophysical activity due to ligand ionophore effects, whereas riboflavin and chlorophyll derivatives generally maintain activity when coordinated in stable trivalent lanthanide systems [27,28]. This indicates a trend: stable Ln^3+^ complexes typically preserve or enhance PS behavior, while uncontrolled ionophore interactions may compromise PDT efficacy.

Natural products such as riboflavin, curcumin, and chlorophyll derivatives have also been successfully integrated into UCNP platforms, demonstrating comparable ROS yields and improved biocompatibility. The details will be discussed in Section 4.1. Rose Bengal (RB) is a halogenated xanthene dye and another representative synthetic PS with strong absorption in the visible region (around 550–560 nm) with high singlet oxygen efficiency, making it a benchmark in PDT research [29]. However, RB is not the target of this manuscript, which centers on natural product PSs and their integration with lanthanide-based systems.

**Table 1:** Examples of upconversion nanoparticle (UCNP) systems for PDT.

Year	Topic	Type	Lanthanide	PS	Consequence	Reference
2011	Magnetic and fluorescent Gd_2_O_3_: Yb^3+^/Ln^3+^ nanoparticles for simultaneous upconversion luminescence/MR dual-modal imaging and NIR-induced photodynamic therapy	UCNPs	Gd_2_O_3_/Yb^3+^/Er^3+^	Zinc phthalocyanine (ZnPc) PDT light dose: 980 nm NIR laser irradiation Excitation (λ_ex_): 980 nm Absorption (λ_abs_): 980 nm (Yb^3+^ sensitizer) Absorption maximum (λ_abs max_): 980 nm Emission (λ_em_): 650 nm Singlet oxygen quantum yield (Φ_Δ_): ~0.52	A multifunctional nanoplatform developed by the combination of Yb^3+^, Er^3+^/Tm^3+^, and Gd^3+^ in a Gd_2_O_3_ host lattice, which achieved the upconversion luminescence and generation of ROS with MRI contrast enhancement for imaging-guided PDT	[30]
2014	Lanthanide-doped upconversion nanoparticles electrostatically coupled with photosensitizers for near-infrared-triggered photodynamic therapy	UCNPs	LiYF_4_:Yb^3+^/Er^3+^	β-carboxyphthalocyanine zinc (ZnPc-COOH) PDT light dose: 980 nm NIR laser irradiation, 0.5–1.0 W/cm^2^ for 5–10 min Excitation (λ_ex_): 980 nm Absorption (λ_abs_): 980 nm (Yb^3+^ absorption band) Absorption maximum (λ_abs max_): 980 nm Emission (λ_em_): 540 nm, 650 nm Singlet oxygen quantum yield (Φ_Δ_): 0.63 ± 0.05 (ZnPc-COOH coupled UCNPs)	LiYF_4_ doped with Yb^3+^, Er^3+^ UCNPs electrostatically coupled with ZnPc-COOH for NIR-triggered PDT, which achieved 96.3% energy-transfer efficiency and strong singlet oxygen generation under 980 nm excitation	[31]
2014	An upconversion nanoparticle–zinc phthalocyanine-based nanophotosensitizer for photodynamic therapy	UCNPs	NaYF^4^:Yb^3+^/Er^3+^	ZnPc PDT light dose: 980 nm NIR laser Irradiation, 0.39 W/cm^2^ Excitation (λ_ex_): 980 nm Absorption (λ_abs_): 980 nm (Yb^3+^ sensitizer) Absorption maximum (λ_abs max_): 980 nm Emission (λ_em_): 650 nm Singlet oxygen quantum yield (Φ_Δ_): 0.52	UCNPs-ZnPc was developed to perform image-guided PDT and caused a liver tumor inhibitory ratio of approximately 80.1%	[32]
2014	Amplifying the red emission of upconverting nanoparticles for biocompatible clinically used prodrug-induced photodynamic therapy	UCNPs	NaYF_4_: Yb^3+^/Er^3+^	Protoporphyrin IX (PpIX) conversion from 5-aminolevulinic acid (ALA) PDT light dose: 980 nm NIR laser irradiation, ≤0.5 W/cm^2^ Excitation (λ_ex_): 980 nm Absorption (λ_abs_): 980 nm (Yb^3+^ sensitizer) Absorption maximum (λ_abs max_): 980 nm (Yb^3+^ absorption peak) Emission (λ_em_): 540 nm, 650–670 nm Singlet oxygen quantum yield (Φ_Δ_): Φ_Δ_ not reported	UCNP-PDT was developed, which possessed a high absolute upconversion quantum yield of 3.2% with NIR irradiation, using red light irradiation in a deep tumor setting in vivo	[33]
2015	A core-shell-shell nanoplatform upconverting near-infrared light at 808 nm for luminescence imaging and photodynamic therapy of cancer	UCNPs	NaYbF_4_:Nd^3+^, NaGdF_4_:Yb^3+^/Er^3+^	Chlorin (Ce6) PDT light dose: 808 nm NIR laser irradiation Excitation (λ_ex_): 808 nm Absorption (λ_abs_): 808 nm (Nd^3+^ sensitizer) Absorption maximum (λ_abs max_): 808 nm Emission (λ_em_): 540 nm, 650 nm Singlet oxygen quantum yield (Φ_Δ_): 0.60	The UCNP system loaded with PS was developed, which successfully generated ROS to effectively kill the intrinsic tumor or the center of large tumors through PDT	[34]
2015	UV-emitting upconversion-based TiO_2_ photosensitizing nanoplatform: near-infrared light mediated in vivo photodynamic therapy via mitochondria-involved apoptosis pathway	UCNPs	NaYF_4_:Yb^3+^/Tm^3+^, NaGdF_4_:Yb^3+^	TiO_2_ shell PDT light dose: 980 nm NIR laser irradiation Excitation (λ_ex_): 980 nm Absorption (λ_abs_): 980 nm (Yb^3+^ sensitizer) Absorption maximum (λ_abs max_): 980 nm Emission (λ_em_): 365 nm Singlet oxygen quantum yield (Φ_Δ_): 0.48	The UCNPs@TiO_2_ NCs were developed, which endocytosed the cancer cells to generate intracellular ROS, decreasing the mitochondrial membrane potential to release cytochrome c into the cytosol and activating caspase 3 to induce cancer cell apoptosis	[35]
2015	The effect of coatings on the affinity of lanthanide nanoparticles to MKN45 and HeLa cancer cell and improvement in photodynamic therapy efficiency	UCNPs	NaYF_4_: Sc^3+^/Yb^3+^/Er^3+^, NaYF_4_: Yb^3+^/Tm^3+^	Protoporphyrin IX (PpIX) conversion from 5-aminolevulinic acid (ALA) PDT light dose: 980 nm NIR laser irradiation Excitation (λ_ex_): 980 nm Absorption (λ_abs_): 980 nm (Yb^3+^ sensitizer) Absorption maximum (λ_abs max_): 980 nm Emission (λ_em_): 540–550 nm, 650–660 nm Singlet oxygen quantum yield (Φ_Δ_): Φ_Δ_ not reported	The amino-modified NaYF_4_ (Sc/Yb/Er) and NaYF_4_(Yb/Tm)-based systems were developed, which enhanced the PDT efficiency against deeper cancer cells of near-infrared light	[36]
2016	Facile assembly of functional upconversion nanoparticles for targeted cancer imaging and photodynamic therapy	UCNPs	NaYF_4_: Yb^3+^/Er^3+^	RB PDT light dose: 980 nm NIR laser irradiation Excitation (λ_ex_): 980 nm Absorption (λ_abs_): 980 nm (Yb^3+^ sensitizer) Absorption maximum (λ_abs max_): 980 nm Emission (λ_em_): 540 nm, 650 nm Singlet oxygen quantum yield (Φ_Δ_): 0.55	PDT nanocomposites functionalized for cancer targeting were developed by coating the UCNPs with a silica layer encapsulating the PS and bioconjugation to antibodies through a bifunctional fusion protein consisting of a solid-binding peptide linker genetically fused to *Streptococcus* Protein G, which generated intracellular ROS and induced NIR-triggered phototoxicity to suppress cancer cell growth in vitro	[37]
2016	A versatile imaging and therapeutic platform based on dual-band luminescent lanthanide nanoparticles toward tumor metastasis inhibition	UCNPs	LiYF_4_: Nd^3+^/Yb^3+^/Er^3+^	RB PDT light dose: 808 nm NIR laser irradiation Excitation (λ_ex_): 808 nm Absorption (λ_abs_): 808 nm (Nd^3+^ sensitizer) Absorption maximum (λ_abs max_): 808 nm Emission (λ_em_): 540 nm (Er^3+^), 1050 nm (Yb^3+^) Singlet oxygen quantum yield (Φ_Δ_): Φ_Δ_ not reported	The Nd^3+^-sensitized lanthanide nanoparticles were activated with covalently linked RB for PDT, and the NIR emission from Yb^3+^ enabled simultaneous imaging with 67% tumor inhibition rate, minimized thermal side effects, such as alleviated liver and spleen burdens, reduced liver injury, and markedly suppressed pulmonary and hepatic metastases	[29]
2016	Upconversion nanophotosensitizer targeting into mitochondria for cancer apoptosis induction and cyt c fluorescence monitoring	UCNPs	NaYF_4_:Yb^3+^/Er^3+^	Triphenylphosphine (TPP) PDT light dose: 980 nm ^3+^NIR laser irradiation Excitation (λ_ex_): 980 nm Absorption (λ_abs_): 980 nm (Yb^3+^ sensitizer in UCNPs) Absorption maximum (λ_abs max_): 980 nm Emission (λ_em_): 540 nm, 650 nm Singlet oxygen quantum yield (Φ_Δ_): 0.45	TPP-UC(PS) was developed, which caused serious mitochondrial matrix swelling by facilitating the in situ detection of cyt c for apoptosis	[38]
2017	Precise photodynamic therapy of cancer via subcellular dynamic tracing of dual-loaded upconversion nanophotosensitizers	UCNPs	LiYF_4_:Yb^3+^/Er^3+^	RB and ZnPc PDT light dose: 980 nm ^3+^NIR laser irradiation Excitation (λ_ex_): 980 nm Absorption (λ_abs_): 980 nm ^3+^(Yb^3+^ sensitizer in UCNPs) Absorption maximum (λ_abs max_): 980 nm Emission (λ_em_): 540 nm, 650 nm Singlet oxygen quantum yield (Φ_Δ_): 0.45–0.5	The UCNPs-PS developed by the combination of poly(allylamine)-modified RB, and ZnPc, which provided an intracellular trafficking of organelle-targeted, leading to the apoptosis of the cancer cells induced by PDT	[39]
2017	Highly emissive dye-sensitized upconversion nanostructure for dual-photosensitizer photodynamic therapy and bioimaging	UCNPs	NaGdF_4_: Yb^3+^/Er^3+^, NaGdF_4_: Nd^3+^/Yb^3+^	Ce6 and Merocyanine 540 (MC540) PDT light dose: 808 nm NIR laser irradiation Excitation (λ_ex_): 808 nm Absorption (λ_abs_): 808 nm (Nd^3+^ sensitizer and IR-808 dye) Absorption maximum (λ_abs max_): 808 nm Emission (λ_em_): 540 nm, 650 nm Singlet oxygen quantum yield (Φ_Δ_): 0.60–0.65	IR-808-sensitized upconversion nanoparticles were developed by the combination of mesoporous silica and Ce6, MC540 PSs loaded inside through covalent bond and electrostatic interaction, which produced a large amount of ROS and low heating effects for the potential clinical application as an imaging-guided PDT technique	[40]
2018	Near-infrared-triggered antibacterial and antifungal photodynamic therapy based on lanthanide-doped upconversion nanoparticles	UCNPs	LiYF_4_:Yb^3+^/Er^3+^	β-carboxyphthalocyanine zinc (CPZ) PDT light dose: 980 nm NIR laser irradiation, 0.5 W/cm^2^ Excitation (λ_ex_): 980 nm Absorption (λ_abs_): 980 nm (Yb^3+^ sensitizer) Absorption maximum (λ_abs max_): 980 nm (Yb^3+^ absorption peak) Emission (λ_em_): 540 nm, 650 nm Singlet oxygen quantum yield (Φ_Δ_): Φ_Δ_ not reported	UCNPs-CPZ-PVP was developed, which reduced the aggregation, and possessed high anti-infectious activity against multi-drug-resistant bacteria (methicillin-resistant *Staphylococcus aureus* by 4.7 log10 and MDR *Escherichia coli* by 2.1 log10) post-aPDT (at 50 μg mL^−1^ UCNPs-CPZ-PVP with 0.5 W cm^−2^ 980 nm light)	[41]
2019	Development of a novel antitumor theranostic platform: a near-infrared molecular upconversion sensitizer for deep-seated cancer photodynamic therapy	UCNPs	NaYF_4_:Yb^3+^/Er^3+^	FUCP-1 PDT light dose: 980 nm NIR laser irradiation Excitation (λ_ex_): 980 nm Absorption (λ_abs_): 980 nm (Yb^3+^ sensitizer in UCNPs) Absorption maximum (λ_abs max_): 980 nm Emission (λ_em_): 540–650 nm Singlet oxygen quantum yield (Φ_Δ_): 0.62	A one-photon excitation molecular PS (FUCP-1) was developed with excellent photostability and outstanding upconversion luminescence quantum yield (up to 12.6%) for imaging-guided PDT, and it was fully metabolized from the body within 24 h to minimize the systemic toxicity	[42]
2020	Upconversion nanoparticle-induced multimode photodynamic therapy based on a metal–organic framework/titanium dioxide nanocomposite	UCNPs	LiYF_4_: Yb^3+^/Er^3+^/Tm^3+^	Porphyrin-based metal–organic framework (MOF) PDT light dose: 980 nm NIR laser irradiation Excitation (λ_ex_): 980 nm Absorption (λ_abs_): 980 nm (Yb^3+^ in UCNPs) Absorption maximum (λ_abs max_): 980 nm Emission (λ_em_): 365 nm, 450–540 nm Singlet oxygen quantum yield (Φ_Δ_): Φ_Δ_ not reported	A nanoplatform developed by the combination of ultrasmall TiO_2_ nanoparticles with UCNP–MOF heterodimers, which demonstrated strong biocompatibility, efficient ROS generation, and effective cancer cell apoptosis, offering a versatile therapeutic approach adaptable to different PDT requirements and overcoming the limitations	[43]
2020	Construction of multicolor upconversion nanotheranostic agent for in situ cooperative photodynamic therapy for deep-seated malignant tumors	UCNPs	NaYF_4_: Nd^3+^/Yb^3+^/ Er^3+^/Tm^3+^	Indocyanine green (ICG) and additional PSs, Ce6 PDT light dose: 808 nm NIR laser irradiation Excitation (λ_ex_): 980 nm Absorption (λ_abs_): 808 nm (Nd^3+^ sensitizer and ICG dye) Absorption maximum (λ_abs max_): 808 nm Emission (λ_em_): 450 nm, 540 nm, 650 nm Singlet oxygen quantum yield (Φ_Δ_): 0.63	A multicolor UCNP nanotheranostic agent was developed by ICG and loaded with PS, which produced multicolor emissions and achieved strong ROS generation in cell apoptosis for deep-seated tumor PDT	[44]
2021	Near-infrared light-triggered photodynamic therapy and apoptosis using upconversion nanoparticles with dual photosensitizers	UCNPs	NaYF_4_: Yb^3+^/Er^3+^/Nd^3+^, NaYF_4_:Yb^3+^/Nd^3+^	Ce6 and RB PDT light dose: 808 nm NIR laser irradiation for 5 min Excitation (λ_ex_): 980 nm Absorption (λ_abs_): 808 nm (Nd^3+^ sensitizer in UCNPs) Absorption maximum (λ_abs max_): 808 nm Emission (λ_em_): 540 nm, 650 nm Singlet oxygen quantum yield (Φ_Δ_): 0.65	Dual-color emitting Er-doped UCNPs and dual PSs were developed for enhancing PDT to generate a high yield of ROS, which induced immunogenic apoptosis	[45]
2021	Cancer-cell-biomimetic upconversion nanoparticles combining chemo-photodynamic therapy and CD73 blockade for metastatic triple-negative breast cancer	UCNPs	NaYF_4_:Yb^3+^/Er^3+^	RB PDT light dose: 980 nm NIR laser irradiation Excitation (λ_ex_): 980 nm Absorption (λ_abs_): 980 nm (Yb^3+^ sensitizer) Absorption maximum (λ_abs max_): 980 nm Emission (λ_em_): 540 nm (Er^3+^), 650 nm (Er^3+^) Singlet oxygen quantum yield (Φ_Δ_): 0.54	CM@UCNP-RB/PDT was developed by the combination of anti-CD73 antibodies, which demonstrate the synergistic efficacy of chemotherapy and PDT, not only destroying the orthotopic tumors but also preventing abscopal tumor metastasis	[46]
2022	PDT-active upconversion nanoheaters for targeted imaging guided combinatorial cancer phototherapies with low-power single NIR excitation	UCNPs	NaGdF_4_: Yb^3+^/Er^3+^	RB PDT light dose: 808 nm NIR laser irradiation Excitation (λ_ex_): 980 nm Absorption (λ_abs_): 808 nm (Yb^3+^/Er^3+^ UCNPs with RB FRET pairing) Absorption maximum (λ_abs max_): 808 nm Emission (λ_em_): 540 nm (Er^3+^), 650 nm (Er^3+^) Singlet oxygen quantum yield (Φ_Δ_): 0.52	The UCNP@Tf-RB was developed for synergistic PTT-PDT, which caused rapid suppression of tumor with a tumor-growth inhibition index, and demonstrated minimal damage to non-targeted tissues	[47]
2023	Photoswitchable upconversion nanoparticles with excitation-dependent emission for programmed stepwise NIR phototherapy	UCNPs	NaErF_4_:Tm^3+^, NaYF_4_: Yb^3+^/Ho^3+^/Nd^3+^, NaYF_4_:Yb^3+^/Tm^3+^	ZnPc PDT light dose: 980 nm NIR laser irradiation of blue emission for PDT, 808 nm NIR laser irradiation of green emission for imaging, 1550 nm NIR laser irradiation of red emission for NO release Excitation (λ_ex_): 980 nm Absorption (λ_abs_): 980 nm (Yb^3+^), 808 nm (Nd^3+^), 1550 nm (Yb^3+^) Absorption maximum (λ_abs max_): 980 nm, 808 nm, 1550 nm Emission (λ_em_): 450 nm (Tm^3+^), 540 nm (Er^3+^), 650 nm (Er^3+^) Singlet oxygen quantum yield (Φ_Δ_): Φ_Δ_ not reported	A smart “off–on” PDT nanoplatform developed by the combination of UCNP with PS and nitric oxide donors, which worked for the independent activation of imaging and generation of ROS using specific light wavelengths to alleviate tumor hypoxia by reducing oxygen consumption	[27]
2023	Utilizing dual-pathway energy transfer in upconversion nanoconjugates for reinforced photodynamic therapy	UCNPs	NaYF_4_: Nd^3+^/Yb^3+^/Er^3+^	Ce6 and AIEgen (aggregation-induced emission luminogen) PDT light dose: 808 nm NIR laser irradiation Excitation (λ_ex_): 808 nm Absorption (λ_abs_): 808 nm (Nd^3+^ sensitizer) Absorption maximum (λ_abs max_): 808 nm Emission (λ_em_): 540 nm, 650 nm Singlet oxygen quantum yield (Φ_Δ_): 0.60–0.65	UCNP-Ce6/AIEgen was developed and might not only undergo direct lanthanide triplet energy transfer to activate Ce6, but also convert the upconversion luminescence of UCNPs to the light, which activated Ce6 through Förster resonance energy transfer to generate more ROS, leading to tumor cell apoptosis	[28]
2023	Mitochondria-targeting upconversion nanoparticles@MOF for multiple-enhanced photodynamic therapy in hypoxic tumor	UCNPs	NaYF_4_: Nd^3+^/Yb^3+^/Er^3+^	TPP PDT light dose: 808 nm NIR laser irradiation Excitation (λ_ex_): 980 nm Absorption (λ_abs_): 808 nm (Nd^3+^ sensitizer in UCNPs) Absorption maximum (λ_abs max_): 808 nm Emission (λ_em_): 540 nm, 650 nm Singlet oxygen quantum yield (Φ_Δ_): 0.66	TPP-UCNPs@MOF-Pt was developed, which effectively relieved the tumor hypoxia by converting intracellular hydrogen peroxide to oxygen located in mitochondria and elevated the ROS level to enhance PDT efficacy	[48]
2025	Synthesis of mesoporous polydopamine-coated upconversion nanoparticles for dual-enhanced photodynamic and photothermal cancer therapy	UCNPs	NaYF_4_:Yb^3+^/Er^3+^, NaYF_4_:Yb^3+^/Nd^3+^	Ce6 PDT light dose: 808 nm NIR laser irradiation for 5 min Excitation (λ_ex_): 980 nm Absorption (λ_abs_): 808 nm (Nd^3+^ sensitizer in UCNPs) Absorption maximum (λ_abs max_): 808 nm Emission (λ_em_): 650 nm Singlet oxygen quantum yield (Φ_Δ_): Φ_Δ_ not reported	The PDT-PTT UCNPs were developed, which emitted the photoluminescence spectra and absorbed by Ce6 to induce the PDT effect for the generation of ROS in human colorectal adenocarcinoma HT-29 cells	[49]
2025	Programming a multiplex lanthanide nanoparticle for customized cancer treatment with real-time efficiency feedback	UCNPs	NaYF_4_: Nd^3+^/Yb^3+^/ Er^3+^/Ho^3+^	RB PDT light dose: 808 nm and 980 nm NIR laser irradiation Excitation (λ_ex_): 980 nm Absorption (λ_abs_): 808 nm (Nd^3+^), 980 nm (Yb^3+^) Absorption maximum (λ_abs max_): 808 nm, 980 nm Emission (λ_em_): 540 nm, 650 nm, 1050–1150 nm Singlet oxygen quantum yield (Φ_Δ_): 0.58–0.62	LNPs-RB/Pep/cRGD was developed, which induced cell apoptosis, generating caspase-3, and cleaved Cy7.5-containing peptide fragments from LNPs by sequentially programming NIR excitation wavelength	[50]

### 3.2. Upconversion–Downshifting Nanoparticle (UDNP) System

In early studies, this focused on Fe/Mn bimetal-doped ZIF-8, which coated luminescent nanoparticles (Nd^3+^/Yb^3+^/Er^3+^/Ho^3+^), combining dual excitation at 808 nm and 980 nm with emissions at 540 nm, 650 nm, and NIR-II from 1050 to 1150 nm. These systems achieved singlet oxygen yields of 0.55–0.60 and enabled tumor self-enhanced imaging. The mesoporous polydopamine-coated UDNPs (Yb^3+^/Er^3+^/Ho^3+^) loaded with merocyanine 540 demonstrated synergistic dual-dynamic therapy, which possessed Φ_Δ_ 0.61, integrating NIR-II fluorescence imaging and gene-related apoptosis pathways. Recently, integrin-targeted composite UDNPs (Er^3+^/Yb^3+^/Nd^3+^/Gd^3+^) activated at 1530 nm using TCPP as a PS, which produced emissions at 660 nm and 1550 nm, with Φ_Δ_ of 0.62–0.65, and regulated tumor microenvironment redox balance, combining PDT with H_2_S gas therapy. These progressions highlighted the evolution of UDNPs toward deeper tissue penetration, higher ROS generation, and multifunctional therapeutic strategies (Table 2).

### 3.3. Differences Between UCNPs and UDNPs

Based on the findings, UCNPs and UDNPs represent distinct lanthanide-based PDT strategies. UCNPs rely on upconversion luminescence, which is converted into visible or UV emission to activate PS, such as ZnPc and Ce6, under 980 nm or 808 nm NIR excitation, whilst UDNPs integrate dual luminescence, combining upconversion from NIR excitation to visible emission and downshifting from UV/visible excitation to NIR-II emission. In practice, most UDNP systems employ NIR excitation (808, 980, or 1530 nm), offering orthogonal NIR-I/II excitation flexibility. Its benefits include (a) reducing the superior background noise by increasing brightness compared with excitation pulses of the same energy and repetition frequency polarized in one direction [54]; (b) improving spatial resolution by efficiently regulating the two-way photoswitching of spiropyran using dual wavelengths of NIR irradiation [55]; and (c) eliminating far-field confounding caused by unwanted far-field excitation in objective-type total internal reflection fluorescence (TIRF) images to enhance the resolution of PDT images for deeper tissue penetration [56].

**Figure 6:**
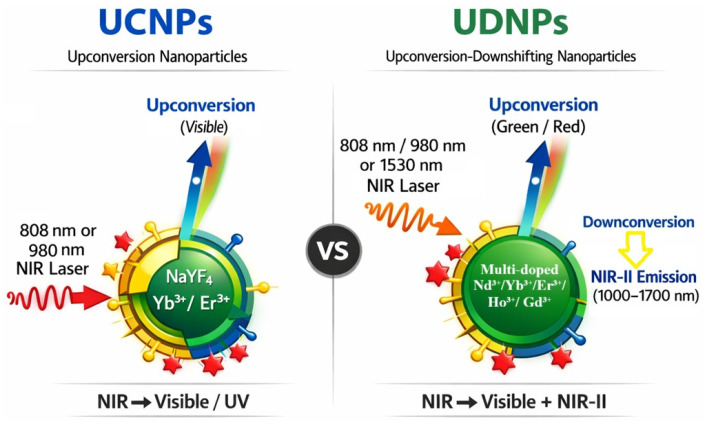
Comparative schematic of UCNPs and UDNPs.Wavy arrows denote NIR laser excitation wavelengths (808 nm, 980 nm, or 1530 nm). The straight blue-to-rainbow arrows represent higher-energy upconversion emission in the visible range (green/red). The open yellow arrow indicates lower-energy downshifting emission in the NIR-II window (1000–1700 nm). The colored concentric rings depict the multi-layered core/shell architectures, where specific chemical dopants (lanthanide ions) are localized in distinct zones to regulate energy transfer.

Generally, UCNPs employ Yb^3+^/Er^3+^ or Yb^3+^/Tm^3+^ dopants, but UDNPs use multi-doped systems, such as Nd^3+^, Yb^3+^, Er^3+^, Ho^3+^, and Gd^3+^ to achieve flexible excitation at 808 nm, 980 nm, or even 1530 nm. UCNPs produce visible or UV bands at 540–650 nm, and 365 nm, whereas UDNPs involve visible and NIR-II emissions from 1000 to 1700 nm (Figure 6). Emission ranges are influenced by several parameters, including (a) sensitizers, e.g., Yb^3+^ and Nd^3+^, which absorb the excitation photons at 980 nm or 808 nm, and the complementary ones which are activations, e.g., Er^3+^, Tm^3+^, and Ho^3+^, which receive the energy from the sensitizers to produce the corresponding emission [57]; (b) nature (thinness or thickness) of the core-shell, where NaYF_4_ is an inert layer to increase radiant efficiency by repairing defects on the surface of UCNPs, whilst the active layer transfers more energy to the activation ions in the nuclear region [58]; size and morphology of nanoparticles in UC which relate to the distribution and luminescence properties, and enhance the dispersibility in a variety of solvents, cellular nontoxicity, in vitro bioimaging, and biocompatibility [59].

According to Table 1 and Table 2, UCNPs achieve singlet oxygen yields of around 0.45–0.66, while UDNPs report 0.55–0.65, which enhances ROS generation and multifunctional therapeutic outcomes. UCNPs are simpler and well established but limited by overheating and single luminescence. The excitation source is at 980 nm for NIR lasers, which causes strong water absorption, these results in local tissue heating to reduce therapeutic efficiency and risks collateral damage, as well as limits the generation of ROS [60]. Thus, UDNPs offer greater penetration depth, multimodal imaging, and synergistic therapies such as PDT combined with gas therapy or photothermal effects, as it has dual excitation wavelengths and reduce overheating, as well as NIR-II emission from 1000 to 1700 nm. The downshifting luminescence produces signals in the NIR-II window, which penetrate deeper into tissue with minimal scattering and absorption [61].

In summary, UCNPs and UDNPs are different approaches depending on the luminescence process, type of lanthanides, range of excitation and emission wavelengths, photosensitizer activation, singlet oxygen yield, advantages, and limitations (Table 3). According to the information collected, UCNPs have established dominance over UDNPs in PDT because of their practicality and efficiency. Compared with UDNPs, which employ flexible NIR excitation (808, 980, or 1530 nm) and dual luminescence pathways. The synthesis is more complex, and their therapeutic applications remain less established. UCNPs also achieve high singlet oxygen yields, Φ_Δ_, from 0.45 to 0.66, and integrate seamlessly with diverse photosensitizers, supporting multifunctional nanoplatforms for imaging, photothermal therapy, and immunotherapy. In contrast, UDNPs offer dual luminescence pathways and orthogonal excitation flexibility, but their synthesis is more complex, and their applications remain largely confined to bioimaging. Hence, UCNPs dominate PDT research, while UDNPs remain promising but less developed for clinical translation. This situation is also applied to the natural product PSs.

## 4. Natural Product PSs

Coumarin, riboflavin, curcumin, chlorophyll derivatives, and hypocrellin are the natural product PSs described in Figure 3. There are some reasons for using natural products instead of porphyrin, chlorin, and phthalocyanine, such as (a) low dark toxicity, which allows for localized, dual-stage selectivity through suitable wavelength absorption, with minimal systemic side effects [62]; (b) biocompatibility, which selectively targets or accumulates in the infection area and degrades or decomposes in the human body [63]; and multimodal actions, which enhance therapeutic efficacy by synergistically generating ROS [64].

### 4.1. Intrinsic Pharmacological Activities

Most natural product PSs derived from Chinese medicinal plants or fungi possess intrinsic pharmacological activities, including anticancer and anti-inflammatory properties, according to traditional Chinese medicine theory (Table 4), which support the therapeutic potential of PDT.

### 4.2. Natural Product PSs in PDT

Apart from the above information, there are some representative examples of natural product PSs for PDT investigated in the past between 2022 and 2026 (Table 5). Coumarin-based systems demonstrated strong absorption in the visible–NIR region, emission ranging from 500 to 770 nm, and singlet oxygen yields of up to 0.839, highlighting its versatility for antibacterial and tumor-targeted PDT [85,86,87,88,89]. Riboflavin is consistently absorbed at 450 nm, emitted at 520 nm, and achieves moderate singlet oxygen yields of about 0.5, proving effective against acne, *Pseudomonas aeruginosa*, HSV-1, and biofilm-associated infections, especially in the combination of adjuvants like potassium iodide or antibiotics [90,91,92,93,94]. Curcumin is absorbed in the blue region at 425 nm, emits green-yellow fluorescence from 500 to 550 nm, and generates lower singlet oxygen yields of about 0.2–0.3, which showed promise in disrupting biofilms, impairing oxidative metabolism, and enhancing antifungal strategies with the usage of chlorhexidine [95,96,97,98,99]. Chlorophyll derivatives are absorbed at 400–450 nm in the Soret band and 660 nm in the Q band, emitted at 670–680 nm, and achieved Φ_Δ_ of 0.5–0.6, supporting applications in cancer therapy and melanoma treatment [100,101,102,103,104]. Hypocrellin derivatives stood out with dual absorption peaks between 450–470 nm and 620–640 nm, emission at 650–670 nm, and high singlet oxygen yields of 0.6–0.8. Notably, the caged-hypocrellin system is absorbed at 480 nm, emitted at 610–620 nm, and achieves Φ_Δ_ of 0.55–0.60, effectively reducing fungal burden in *Candida auris* infections while modulating immune responses [105,106,107,108,109]. These findings underscore the evolution of PDT PSs toward higher efficiency, broader antimicrobial coverage, and enhanced biocompatibility. Chlorophyll derivatives and hypocrellin offer particularly strong PDT therapeutic potential in the treatment.

## 5. Natural Product-Based Upconversion PSs in PDT

Recently, scientists have drawn attention to the lanthanide-based systems, e.g., complexes or UCNPs, which are integrated with natural products, and have emerged as promising platforms for further improving the performance of PDT. As lanthanide ions have unique photophysical properties that combine with the bioactivity of natural product PSs, these innovative hybrid constructs offer enhanced luminescence, energy transfer efficiency, and therapeutic potential of PDT in diverse biomedical applications. Thus, current research emphasizes the photophysical properties, synergistic interactions with natural products, and translational promise in PDT.

### 5.1. Photophysical Properties

Lanthanide ions have the heavy-atom effect, which facilitates ISC from the PS singlet state to the triplet state, drastically boosting ROS generation. They exhibit high two-photon absorption cross-section combined with NIR emission or excellent ^1^O_2_ generation. The very large intersystem crossing efficiency is induced by the heavy rare-earth elements, like Tb^3+^, Eu^3+^, and Gd^3+^ [110]. For example, a solid metal complex confirmed with the optical activity from electronic absorption spectra at wavelengths from 280 to 390 nm and intense phosphorescence bands up to 830 nm. This enables PDT for apoptosis of cancer cells by converting triplet oxygen in the tissues into reactive singlet oxygen through the heavy-atom effect. The energy transfer associated with these phosphorescence bands correlates with strong photodynamic activity [111].

Meanwhile, lanthanide ions exhibit sharp emission bands arising from characteristic f–f transitions of 4f electrons. This luminescence underpins applications in disease diagnosis and biological imaging, particularly within the NIR-II window (1000–1700 nm), which enables deep tissue visualization. These emission properties support PDT by triggering bioresponsive functions in cellular pathways [2,112].

### 5.2. Natural Products Synergy

Coumarin, riboflavin, curcumin, chlorophyll derivatives, and hypocrellins are natural PSs, which demonstrate preferential accumulation at sites of infection through intrinsic targeting mechanisms, thereby sparing normal tissues from unintended effects [113,114,115]. This selective behavior reduces off-target toxicity, while their multifunctional pharmacological activities further enhance PDT efficacy, including anti-inflammatory and anticancer effects, as discussed in Section 4.

These natural PSs gain additional photophysical advantages when combined with lanthanide ions, such as Tb^3+^, Eu^3+^, and Gd^3+^, for PDT. The conjugation of lanthanides with natural PSs, often achieved through non-covalent loading, enables closer molecular interactions and confers high energy-transfer efficiency for effective ROS generation [116]. UCNPs and UDNPs are the systems to exemplify this synergy, allowing activation under NIR excitation and extending PDT applications to deeper tissues with improved therapeutic outcomes, as discussed in Section 3.

### 5.3. Photophysical Mechanisms of Lanthanide Ions in Natural Product Photosensitizer Systems

Generally, this is the same as the upconversion as described in Section 2.1. It involves a series of steps, including NIR absorption by UCNPs, spectral overlap with natural products through the excitation, and biological effects. UCNPs are typically lanthanide-doped crystals, such as NaYF_4_:Yb^3+^/Er^3+^ or NaYF_4_:Yb^3+^/Tm^3+^. It has sensitizer ions (Yb^3^ or Nd^3+^), which absorb NIR light from 808 to 980 nm. Upon light irradiation, the energy is transferred to activator ions (Er^3+^/Tm^3+^/Ho^3+^), which emit higher-energy photons in the visible or UV region from blue to red, i.e., anti-Stokes emission [117].

**Figure 7:**
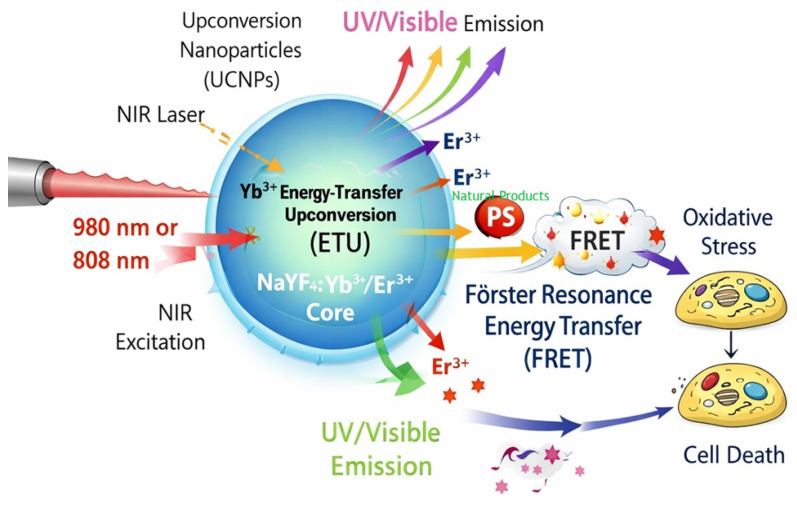
Mechanistic illustration of UCNP-mediated natural product PS PDT. Wavy arrows represent NIR laser excitation inputs (red: 808/980 nm; orange: 808/980/1530 nm). Multi-colored gradient arrows indicate upconversion emissions into the visible spectrum (blue/green/red). The open yellow arrow represents downshifting emission into the NIR-II window (1000–1700 nm). The segmented green and yellow concentric rings illustrate the multi-doped layer distributions of lanthanide activators within the nanoparticle core-shell architecture.

Natural product PSs include coumarin, riboflavin, curcumin, chlorophyll derivatives, and hypocrellin. They absorb UCNP-emitted photons from the ground state to reach an excited singlet state. The electron transition becomes a triplet state through the ISC, and the triplet state electrons in PS transfer energy for the generation of ROS, such as singlet oxygen (^1^O_2_). These ROS induce oxidative damage to the cellular components, e.g., protein DNA [118]. NIR (980 nm or 808 nm) excitation of Yb^3+^ ions triggers ETU to Er^3+^/Nd^3+^, producing UV/visible emissions. The red-region emission overlaps with the natural product PSs’ absorption band, enabling FRET. Excited natural product PS molecules generate ROS, which induce oxidative stress, cell damage, and ultimately cell death (Figure 7).

### 5.4. Research Process

Over recent investigations, the evolution of natural product PSs integrated with lanthanide complexes and UCNPs has been systematically explored to advance PDT. This involves the coumarin–lanthanide complexes [119,120], which demonstrated strong visible emission and high luminescence quantum yield, thereby indicating significant potential for application in PDT. Riboflavin–lanthanide-based systems demonstrated efficient activation under near-infrared or blue light, achieving a singlet oxygen quantum yield of approximately 0.5 and enabling selective phototoxicity against cancer cells [121,122]. Curcumin conjugates consistently employed UCNPs with 980 nm excitation, achieving a singlet oxygen quantum yield of between 0.4 and 0.6, and the latter designs incorporated MnO_2_ shells or MOF-lanthanide agents to enhance tumor responsiveness and synergistic therapy [123,124,125,126,127,128]. Pyropheophorbide a (PPa) conjugated to lanthanide-doped UCNPs achieves a high singlet oxygen quantum yield of between 0.52 and 0.79 through targeted nanoplatforms, integrating tumor microenvironment responsiveness [129,130].

Hypocrellin derivatives also conjugated to lanthanide-doped UCNPs reach a singlet oxygen quantum yield up to 0.79 with orthogonal NIR excitation, while HB–lanthanide complexes improve absorption and singlet oxygen quantum yield of 0.32, and HB–UCNPs possess a singlet oxygen quantum yield of about 0.6 with effective breast cancer cell killing (Table 6) [131,132,133,134,135]. These findings demonstrate the rational design of nanoplatforms combining PSs with UCNPs, lanthanide complexes, or MOFs to enhance singlet oxygen generation, tissue penetration, and therapeutic efficacy, positioning systems as versatile PDT agents for biological applications.

### 5.5. Conventional Use and Lanthanide-Enhanced Systems

By comparing Table 5 and Table 6, these natural product PSs absorb photons in the UV–visible range, undergo singlet excitation, and transition via ISC to the triplet state. The triplet PS transfers energy for the generation of ROS, particularly ^1^O_2_. These ROS induce oxidative damage to proteins, lipids, and DNA, leading to cell apoptosis and necrosis. However, the limitations remain, including shallow tissue penetration, photobleaching, and relatively low singlet oxygen quantum yields.

Lanthanide-enhanced systems overcome these drawbacks. UCNPs doped with Yb^3+^/Er^3+^ or other Tm^3+^/Er^3+^ lanthanides absorb NIR light and emit visible photons, which activate natural PSs even under deep tissue irradiation. Coumarin–lanthanide complexes exhibit strong visible emission and high quantum yields, riboflavin-UCNP conjugates enable NIR-triggered PDT, and curcumin-UCNP hybrids enhance ROS generation for tumor hypoxia. Similarly, chlorophyll derivatives conjugated to UCNPs achieve targeted NIR PDT, while hypocrellin–lanthanide complexes display red-shifted absorption and improved singlet oxygen yields. These systems provide enhanced luminescence, stability, and therapeutic depth, integrating imaging and therapy in a single platform, deeper tissue penetration, less photobleaching, and high singlet oxygen quantum yields (Table 7).

## 6. Discussion

Lanthanide-based natural product PSs represent a transformative advance in PDT, combining biocompatibility with superior photophysical performance for clinical translation. However, there are some considerations.

### 6.1. Synergistic Mechanisms

The photophysical properties of lanthanide ions are unique, and they synergize with natural product PSs to overcome limitations such as poor solubility, low bioavailability, and shallow tissue penetration. The lanthanide-doped nanoparticles exploit upconversion luminescence, converting deeply penetrating 980 or 808 nm NIR light into visible emission that activates PSs, thereby bypassing the short excitation wavelengths of natural compounds. Similarly, downshifting luminescence produces single lower-energy photons in the NIR-II window (1000–1700 nm), which penetrates deeper into tissue and enhances imaging contrast. This emission is valuable for bioimaging but does not directly excite PSs or generate ROS. Thus, the dual luminescence processes serve complementary roles in the upconversion luminescence, which activates PSs and drives ROS generation for PDT, while downshifting luminescence provides deep-tissue imaging and monitoring.

The lanthanide-based systems act as energy transducers [136], stabilizing natural PSs and extending their therapeutic reach, while simultaneously mitigating solubility and bioavailability issues through nanoparticle encapsulation and surface functionalization [137]. These synergies establish a mechanistic framework where lanthanides transform natural PSs from biocompatible but limited agents into clinically promising tools for deep-tissue PDT.

### 6.2. Effectiveness Comparison

Chemically synthesized PS, such as porphyrins, chlorins, phthalocyanines, BODIPY, and metal–ligand complexes, offer advantages including highly selective, light-activated tumor destruction and the capacity for deep tissue penetration. Several porphyrin derivatives, such as photofrins, have already received FDA approval for clinical use [138]. In contrast, natural product PSs, such as coumarin, riboflavin, curcumin, chlorophyll derivatives, and hypocrellins, provide lower systemic toxicity, rapid metabolic clearance, and reduced risk of long-term side effects compared with synthetic porphyrins or phthalocyanines [139]. These attributes make natural PSs a safer and more biocompatible alternative.

### 6.3. Clinical Translation

The major barriers to translate lanthanide-based natural product PDT systems into clinical practice include toxicity, stability, and regulatory approval from the FDA.

Lanthanides have selective toxicity, which depends on the type of genomic signatures, such as oxidative stress responses, mitochondrial dysfunction, DNA repair, and cell cycle control, as well as metal homeostasis pathways. Understanding these pathway-specific genomic signatures is critical for designing safer lanthanide-based nanoplatforms, guiding dose optimization, and ensuring biocompatibility in clinical translation [140].

The stability of lanthanides are highly reliant on thermodynamic, kinetic, and ligand design. When the atomic number increases, lanthanide contraction occurs, and the ionic radius of the lanthanide (III) ion decreases. This decreasing size results in higher charge density, leading to increased stability of complexes [141]. It is also highly thermodynamically stable in aqueous solution at physiological pH [142]. Natural product PSs can serve as ligands, but their design must minimize steric hindrance, optimize electron-donating ability, and control counterion retention to stabilize high-oxidation-state lanthanide complexes [143]. Typically, for a lanthanide-based natural product PDT system to gain FDA regulatory approval, it must go through the same rigorous pathway as any other drug, including preclinical studies, Investigational New Drug (IND) Application, clinical trials (Phases I–III), New Drug Application (NDA), and post-market surveillance (Phase IV) [144,145,146]. Up to the present, the FDA has approved the lanthanide radioisotope lutetium-177 in cancer therapies, Pluvicto (lutetium Lu 177 vipivotide tetraxetan) for metastatic castration-resistant prostate cancer, and Lutathera (lutetium Lu 177 dotatate) [147,148,149]. Overcoming these barriers is essential to bridge preclinical promise with clinical reality, enabling lanthanide-based natural products for PDT systems to evolve into viable therapeutic options.

## 7. Conclusions

Natural product-based UCNP PSs represent an innovative strategy in PDT, offering a unique convergence of biocompatibility and advanced photophysical performance compared with conventional therapy. The incorporation of lanthanide ions enables dual luminescence processes, such as UCNPs and UDNPs. These systems help overcome key limitations of natural PSs, including poor solubility, low bioavailability, and shallow tissue penetration. The visible upconversion emission overlaps with PS absorption and drives ROS. NIR-II downshifting is imaging only.

Recent advances in multifunctional nanoplatforms with the support of nanotechnology, which possess the versatility of lanthanide-based PDT, demonstrate not only improved therapeutic efficacy but also the potential for multimodal actions for PDT strategies. Natural product PSs with the advantages of less systemic toxicity and rapid clearance are safer alternatives to chemically synthesized PSs. However, there are significant barriers that remain for clinical translation, including the selective toxicity and stability of lanthanide ions in UCNPs and UDNPs, as well as their complexes. UCNPs are the dominant strategy in PDT, since it has a longer track record, with numerous preclinical studies demonstrating tumor inhibition and antibacterial activity. FDA approval is the next milestone to be achieved, which requires rigorous preclinical evaluation, IND submission, phased clinical trials, and post-market surveillance. Current successful examples, such as the approval of lutetium-177 radiopharmaceuticals (Pluvicto and Lutathera), serve as benchmarks or references for the lanthanide-based natural product PS in UCNP or UDNP systems.

Thus, natural product-based UCNP systems embody a transformative therapeutic paradigm if they can address the above issues to progress from preclinical promise to clinically viable therapies.

## 8. Future Aspects

Chemically, flavins such as riboflavin are benzopteridines, belonging to the broader pteridine family. Pteridines, including conjugated forms like folic acid and unconjugated forms like pterin, also display photochemical activity relevant to PDT. Recent advances in the photochemistry of pterins have achieved efficient photooxidation and singlet oxygen generation under UV–visible irradiation [150]. These findings highlight the broader relevance of pteridine derivatives as natural PSs. The significant barriers of lanthanide natural product-based PDT systems should be overcome. Future work should prioritize improving the pharmacological stability of natural product PSs within UCNP constructs, ensuring reproducibility of ROS yields, and bridging preclinical promise with clinical translation. There has been an innovative approach in recent years, but the information is still limited, which applies to double-natural PSs. For example, Zhao et al. reported Er/Tm-enriched orthogonal UCNPs that simultaneously activated distinct emission channels to drive photodynamic immunomodulation, achieving suppression of tumor metastasis [151]. These strategies make the multi-PS integration and orthogonal excitation pathways overcome current limitations of single-PS UCNP systems and become more efficient and clinically translatable PDT.

## Figures and Tables

**Table 2:** Examples of upconversion–downshifting nanoparticle (UDNP) systems for PDT.

Year	Topic	Type	Lanthanide	PS	Consequence	Reference
2023	Fe/Mn bimetal-doped ZIF-8-coated luminescent nanoparticles with up/downconversion dual-mode emission for tumor self-enhanced NIR-II imaging and catalytic therapy	UDNPs	Nd^3+^/Yb^3+^/ Er^3+^/Ho^3+^	RB PDT light dose: 808 nm and 980 nm NIR laser irradiation Excitation (λ_ex_): 1530 nm Absorption (λ_abs_): 808 nm (Nd^3+^), 980 nm (Yb^3+^) Absorption maximum (λ_abs max_): 808 nm, 980 nm Emission (λ_em_): 540 nm, 650 nm, 1050–1150 nm Singlet oxygen quantum yield (Φ_Δ_): 0.55–0.60	LDNPs@Fe/Mn-ZIF-8 was developed, which decreased the bandgap of the ZIF-8 photosensitizer from 5.1 to 1.7 eV, expanding the excitation threshold of ZIF-8 to the visible light region (∼650 nm), as well as endowed the nanosystems with tumor self-enhanced NIR-II imaging function	[51]
2026	Multi-band excitation of NIR-II lanthanide nanoparticles via multiple-dye cascade sensitization	UDNPs	Nd^3+^/Yb^3+^/Er^3+^ /Tm^3+^/Ho^3+^	Tropolone, Cyanine-3, IR806 (sensitizers/cascade dyes) PDT light dose: not reported Excitation (λ_ex_): Multi-band (270–1030 nm via dye cascade; 808 nm and 1530 nm NIR excitation) Absorption (λ_abs_): 270–1030 nm (UV–Vis–NIR) Absorption maximum (λ_abs max_): ~270 nm (Tropolone), ~550 nm in Vis (Cyanine-3), ~806 nm in NIR (IR806) Emission (λ_em_): 1060 nm to 1525 nm (NIR-II emission) Singlet oxygen quantum yield (Φ_Δ_): Φ_Δ_ not reported	The multi-band dye cascade sensitization greatly expanded the excitation window of lanthanide-doped nanoparticles to have a strong and tunable NIR-II emission for deep-tissue bioimaging and multimodal PDT synergy	[52]
2026	Integrin-targeted delivery of redox homeostasis regulating lanthanide-based composite nanoplatform for deep self-enhanced photodynamic/H_2_S gas synergistic therapy via 1530 nm activation	UDNP	Er^3+^/Yb^3+^/ Nd^3+^/Gd^3+^	Meso-tetra(4-carboxyphenyl) porphine (TCPP) PDT light dose: 1530 nm NIR laser irradiation Excitation (λ_ex_): 980 nm and 1530 nm Absorption (λ_abs_): 1530 nm (Er^3+^ sensitizer) Absorption maximum (λ_abs max_): 1530 nm Emission (λ_em_): 660 nm, 1550 nm Singlet oxygen quantum yield (Φ_Δ_): 0.62–0.65	UDNPs@MOF:DATS@cRGD-PEG was developed, which regulated the levels of dissolved oxygen and GSH in the TME, improving ROS generation, as well as producing a gas therapy (GT) effect for enhancing the PDT/GT synergistic effect of deep-seated tumors	[53]

**Table 3:** Comparison of UCNP vs. UDNP systems.

Feature(s)	UCNPs	UDNPs
Luminescence process	NIR to visible or UV light	Dual pathways: upconversion from NIR (808, 980, 1530 nm) to visible light (NIR-I) plus downshifting from UV/visible excitation to NIR-II emission
Type of lanthanides (sensitizers and activators)	Yb^3+^ (sensitizer), Er^3+^/Tm^3+^ (activators)	Multi-doped: Nd^3+^/Yb^3+^/Er^3+^/Ho^3+^/Gd^3+^
Range of excitation and emission wavelengths	980 nm (Yb^3+^) or 808 nm (Nd^3+^) Visible (green/red from 540 to 650 nm) or UV (365 nm)	808 nm, 980 nm, and extend to 1530 nm, Visible (540–650 nm) and NIR-II (1000–1700 nm)
Photosensitizer activation	ZnPc, Ce6	RB, MC540, TCPP
Singlet oxygen yield	0.45–0.66	0.55–0.65
Advantages	Low cytotoxicity	Orthogonal excitation without overheating
Limitations	Overheating	More complex synthesis

**Table 4:** Intrinsic pharmacological activities of natural product PSs.

Source	Natural Product PS	Traditional Chinese Medicine Theory	Function	Mechanism
*Chaihu* (Bupleurum), *Qianhu* (Peucedanum) [64]	Coumarin	Blood circulation and resolving inflammation [65]	Anticancer	Suppress PI3K-AKT-mTOR pathway activity across cancers, and modulate NF-κB and MAPK pathways to induce apoptosis [66], inhibition of tumor cell proliferation, modulation of oxidative stress, and inhibition of angiogenesis and metastasis [67]
			Anti-inflammatory	Inhibit the PGE2 and NO, as well as pro-inflammatory cytokines TNF-α, IL-6, and IL-1β production [68]
Hawthorn (Crataegus) [69], Goji berries (Lycium barbarum and Lycium chinense) [70]	Riboflavin	Support the spleen and stomach systems for managing the body’s energy and blood metabolism [71]	Anticancer	Inhibit cancer cell proliferation through p21-mediated G1/S phase arrest and suppress DNA replication by downregulating the Mcm helicase complex [72]
			Anti-inflammatory	Inhibit the pro-inflammatory cytokines TNF-α, IL-1β, and CALP, with an increase in the anti-inflammatory cytokine IL-10 [73]
Rhizome of *Curcuma longa* L. [74]	Curcumin	Remove blood stasis, promote blood circulation, and relieve pain [75]	Anticancer	Inhibits the activation of signaling pathways, including the MAPK, PI3K/Akt, and NF-kB pathways, resulting in the induction of apoptosis, prevention of proliferation, and suppression of cancer [76]
			Anti-inflammatory	Targets various inflammatory mediators such as cyclooxygenase-2, inducible nitric oxide synthase, and nuclear factor κB (NF-κB), thereby attenuating the release of proinflammatory and profibrotic cytokines, and suppressing chronic production of free radicals for acute inflammatory reactions [77]
*Can Ye* (mulberry leaf) and *Can Sha* (silkworm excreta) [78]	Chlorophyll derivatives	Clear heat, resolve dampness, detoxify, harmonize the stomach and liver [78]	Anticancer	Inhibit cellular glutathione (GSH), increase ROS levels, and subsequently upregulate the HSP70 protein in response to heightened oxidative stress for anticancer effects [79]
			Anti-inflammatory	Downregulate the p38 MAPK and NFκB signaling pathways to reduce the levels of pro-inflammatory cytokines TNF-α and IL-6 for antihyperalgesic, anti-inflammatory, and antiarthritic effects [80]
Parasitic fungus (hypocrella bambusae) [81]	Hypocrellin	Treat stomach pain, rheumatoid arthritis, limb numbness, anemia, and headache [82]	Anticancer	Inhibits cancer cell proliferation and migration, which promotes apoptosis in vitro by targeting the AKT/STING signaling pathway [83]
			Anti-inflammatory	Suppresses the inflammatory responses mediated by the NLRP3 inflammasome, and blocks the NLRP3-NEK7 interaction [84]

**Table 5:** Representative examples of natural product PSs in PDT.

Year	Topic	PS	Parameter(s)	Consequence	Reference
2022	A coumarin-based fluorescent probe for NIR imaging-guided photodynamic therapy against S. aureus-induced infection in mouse models	Coumarin	PDT light dose: 660 nm NIR laser irradiation Excitation (λ_ex_): 980 nm Absorption (λ_abs_): 660 nm Absorption maximum (λ_abs max_): 450 nm Emission (λ_em_): 670 nm Singlet oxygen quantum yield (Φ_Δ_): Φ_Δ_ not reported	A coumarin-based fluorescent probe was developed, which was effectively triggered and facilitated the exploration of the next generation of antibacterial materials for PDT	[85]
2023	A near-infrared light-activatable Ru(II)-coumarin photosensitizer active under hypoxic conditions	Ru(II)–coumarin complex	PDT light dose: 740 nm NIR irradiation Excitation (λ_ex_): 980 nm Absorption (λ_abs_): Visible–NIR region Absorption maximum (λ_abs max_): Coumarin at 450 nm, and extend absorption into the 740 nm NIR region Emission (λ_em_): 770 nm Singlet oxygen quantum yield (Φ_Δ_): 0.60–0.70	The novel Ru(II)–coumarin conjugate was developed, which exhibited water solubility and dark stability in biological media to generate ROS, hampering its therapeutic efficacy against hypoxic solid tumors in PDT	[86]
2024	Synthesis of coumarin-based photosensitizers for enhanced antibacterial type I/II photodynamic therapy	Coumarin and AIEgen	PDT light dose: not reported Excitation (λ_ex_): 980 nm Absorption (λ_abs_): not reported Absorption maximum (λ_abs max_): not reported Emission (λ_em_): not reported Singlet oxygen quantum yield (Φ_Δ_): 0.839	A coumarin-based aggregation-induced emission luminogen (AIEgen) was developed, which possessed broad-spectrum fluorescence imaging and exhibited excellent biocompatibility for eliminating pathogenic microorganisms	[87]
2024	Effect of biphenyl derivative of coumarin compound photodynamic therapy on the expression of carcinoma-associated genes	Coumarin and ZnPc	PDT light dose: Visible light, and ZnPc excitation at 670 nm Excitation (λ_ex_): 980 nm Absorption (λ_abs_): Coumarin derivatives in UV–visible range from 350 to 450 nm, ZnPc in the red/NIR region at 670 nm Absorption maximum (λ_abs max_): Coumarin from 420 to 450 nm, ZnPc at 670 nm Emission (λ_em_): Coumarin in the blue–green region from 480 to 500 nm, ZnPc emits in the red/NIR from 680 to 690 nm Singlet oxygen quantum yield (Φ_Δ_): Φ_Δ_ not reported	The biphenyl derivative of coumarin compounds was developed together with ZnPc in PDT, which significantly reduced cell viability and induced apoptosis at a rate of 53%	[88]
2025	Heavy-atom free bodipy-borafluorene photosensitizer decorated with coumarin antenna selectively staining endoplasmic reticulum for application in PDT	Coumarin and BODIPY	PDT light dose: Green light irradiation at 520 nm Excitation (λ_ex_): 980 nm Absorption (λ_abs_): Visible region Absorption maximum (λ_abs max_): BODIPY at 520 nm, coumarin from 350 to 450 nm Emission (λ_em_): Green–red region from 540 to 600 nm Singlet oxygen quantum yield (Φ_Δ_): 0.70–0.80	A novel heavy-atom-free BODIPY with coumarin was developed, which localized selectively in the endoplasmic reticulum with good biocompatibility, small dark cytotoxicity, and efficient singlet oxygen generation, causing cellular death in PDT	[89]
2024	Topical riboflavin versus 5-aminolevulinic acid photodynamic therapy for the treatment of mild to moderate acne: a split-face randomized study	Riboflavin	PDT light dose: 450 nm, 3 sessions for one-week intervals Excitation (λ_ex_): 808 nm Absorption (λ_abs_): Broad in the UV–blue region Absorption maximum (λ_abs max_): 450 nm Emission (λ_em_): 520 nm Singlet oxygen quantum yield (Φ_Δ_): 0.5	Riboflavin-PDT significantly reduced non-inflammatory and inflammatory acne in treating mild to moderate acne vulgaris with fewer in-treatment and post-treatment adverse events	[90]
2024	Antimicrobial photodynamic effect of the photosensitizer riboflavin, alone and in combination with colistin, against pan-drug-resistant *Pseudomonas aeruginosa* clinical isolates	Riboflavin	PDT light dose: 450 nm, ~1000 mW/cm^2^, fluoresces ~30–60 J/cm^2^ Excitation (λ_ex_): 808 nm Absorption (λ_abs_): Broad absorption in the UV–blue region Absorption maximum (λ_abs max_): 450 nm Emission (λ_em_): 520 nm Singlet oxygen quantum yield (Φ_Δ_): 0.5	Riboflavin-PDT was successfully combined with antibiotics, such as colistin, for the treatment of difficult-to-treat *P. aeruginosa* infections	[91]
2025	Improvement of antibacterial potency of riboflavin-mediated photodynamic therapy by potassium iodide against *Aggregatibacter actinomycetemcomitans* biofilm formed on orthodontic miniscrews: an in vitro study	Riboflavin	PDT light dose: 1000–1400 mW/cm^2^, 60–80 J/cm^2^, for 1 min Excitation (λ_ex_): 808 nm Absorption (λ_abs_): Broad absorption in the UV–blue region Absorption maximum (λ_abs max_): 450 nm Emission (λ_em_): 520 nm Singlet oxygen quantum yield (Φ_Δ_): 0.5	Riboflavin-PDT combined with potassium iodide, which effectively reduced *A. actinomycetemcomitans* biofilms formed on orthodontic miniscrews	[92]
2025	Targeting HSV-1 glycoprotein D through riboflavin-mediated photodynamic therapy: insights from bioinformatics analysis and in vitro evaluation	Riboflavin	PDT light dose: 450 nm, 30–60 J/cm^2^ Excitation (λ_ex_): 808 nm Absorption (λ_abs_): Broad absorption in the UV–blue region Absorption maximum (λ_abs max_): 450 nm Emission (λ_em_): 520 nm Singlet oxygen quantum yield (Φ_Δ_): 0.5	Riboflavin-PDT was successfully engaged with glycoprotein D, demonstrating a high binding affinity, which effectively suppressed HSV-1 replication	[93]
2025	Effect of riboflavin and blue light-emitting diode irradiation on microbial inactivation and the physicochemical properties of betel leaves	Riboflavin	PDT light dose: 450 nm, 30–60 J/cm^2^ Excitation (λ_ex_): 980 nm Absorption (λ_abs_): Broad absorption in the UV–blue region Absorption maximum (λ_abs max_): 450 nm Emission (λ_em_): 520 nm Singlet oxygen quantum yield (Φ_Δ_): 0.5	Riboflavin-PDT significantly reduced microbial populations by up to 5.3 log CFU/g for *E. coli* and 6.2 log CFU/g for *L. innocua* on leaf surfaces for enhancing microbial safety and promoting phytochemical quality in betel leaves	[94]
2024	Photodynamic therapy with curcumin and near-infrared radiation as an antitumor strategy to glioblastoma cells	Curcumin	PDT light dose: 450 nm, 2 J/cm^2^ Excitation (λ_ex_): 980 nm Absorption (λ_abs_): 420–430 nm in the blue region Absorption maximum (λ_abs max_): 425 nm Emission (λ_em_): 500–550 nm in the green-yellow fluorescence Singlet oxygen quantum yield (Φ_Δ_): 0.20–0.30	Curcumin-PDT increased the levels of ROS, decreased cell proliferation, and enhanced cytotoxicity with cell death by autophagy and necrosis	[95]
2024	Assessing the effects of curcumin and 450 nm photodynamic therapy on oxidative metabolism and cell cycle in head and neck squamous cell carcinoma: an in vitro study	Curcumin	PDT light dose: 450 nm, 15 J/cm^2^ for 60 s Excitation (λ_ex_): 980 nm Absorption (λ_abs_): 420–430 nm in the blue region Absorption maximum (λ_abs max_): 425 nm Emission (λ_em_): 500–550 nm in the green-yellow fluorescence Singlet oxygen quantum yield (Φ_Δ_): 0.2–0.3	Curcumin-PDT caused an oxidative phosphorylation metabolism impairment, which induced an uncoupling between respiration and energy production, leading to increased oxidative damage, a cellular growth and viability reduction, and a cell cycle block in the G1 phase for head and neck squamous cell carcinoma	[96]
2024	In vitro effect of photodynamic therapy with curcumin in combination with photobiomodulation therapy at 660 nm on the viability of human gingival fibroblasts	Curcumin	PDT light dose: 430 nm, 15 J/cm^2^ and PBMT at 660 nm Excitation (λ_ex_): 980 nm Absorption (λ_abs_): 420–430 nm in the blue region Absorption maximum (λ_abs max_): 425 nm Emission (λ_em_): 500–550 nm in the green-yellow fluorescence Singlet oxygen quantum yield (Φ_Δ_): 0.2–0.3	PBMT laser irradiation was under 660 nm after curcumin-PDT, and the viability of HGFs would increase by increasing the frequency of irradiation	[97]
2024	Utilizing the photodynamic properties of curcumin to disrupt biofilms in *Cutibacterium acnes*: a promising approach for treating acne	Curcumin	PDT light dose: 450 nm, 15 J/cm^2^ Excitation (λ_ex_): 980 nm Absorption (λ_abs_): 420–430 nm in the blue region Absorption maximum (λ_abs max_): 425 nm Emission (λ_em_): 500–550 nm in the green-yellow fluorescence Singlet oxygen quantum yield (Φ_Δ_): 0.2–0.3	Curcumin-PDT was an alternative approach for the treatment of *C. acnes*, especially in instances of antibiotic-resistant strains and infections related to biofilms	[98]
2025	Chlorhexidine combined with curcumin-mediated photodynamic treatment effectively inhibits biofilm formation by clinical Candida isolates from the oral cavity	Curcumin	PDT light dose: 450 nm, 15 J/cm^2^ Excitation (λ_ex_): 980 nm Absorption (λ_abs_): 420–430 nm in the blue region Absorption maximum (λ_abs max_): 425 nm Emission (λ_em_): 500–550 nm in the green-yellow fluorescence Singlet oxygen quantum yield (Φ_Δ_): 0.2–0.3	Chlorhexidine combined with curcumin-PDT under high-dose irradiation significantly enhanced the biofilm inhibition of *Candida* spp., for a promising antifungal strategy in the management of biofilm-associated infections	[99]
2023	Anticancer effect of chlorophyllin-assisted photodynamic therapy to induce apoptosis through oxidative stress on human cervical cancer	Chlorophyll derivatives	PDT light dose: 660 nm, 10–20 J/cm^2^ Excitation (λ_ex_): 980 nm Absorption (λ_abs_): 400–450 nm (Soret band), 660 nm (Q band) Absorption maximum (λ_abs max_): 660 nm Emission (λ_em_): 670–680 nm Singlet oxygen quantum yield (Φ_Δ_): 0.5–0.6	Chlorophyll-PDT downregulated the phosphorylation of AKT1, increased protein expression levels of the cleaved caspase 8, caspase 9, Bax, and cytochrome C, and suppressed protein expression levels of Bcl-2, pro-caspase 8, and pro-caspase 9 for cervical cancer	[100]
2023	Photodynamic therapy directed to melanoma skin cancer by thermosensitive hydrogel containing chlorophyll A	Chlorophyll A	PDT light dose: 660 nm, 10–20 J/cm^2^ Excitation (λ_ex_): 980 nm Absorption (λ_abs_): 400–450 nm (Soret band), 660 nm (Q band) Absorption maximum (λ_abs max_): 660 nm Emission (λ_em_): 670–680 nm Singlet oxygen quantum yield (Φ_Δ_): 0.5–0.6	Chlorophyll A incorporated into hydrogels serves as a thermosensitive system for topical applications, and when associated with PDT, it exhibited potential against the melanoma cell line	[101]
2025	Photodynamic activation of a novel chlorophyll-enriched green propolis compound triggers apoptosis in renal cell carcinoma	Chlorophyll derivatives	PDT light dose: 570 nm, 10–15 J/cm^2^ Excitation (λ_ex_): 1530 nm Absorption (λ_abs_): 400–450 nm in the blue–green region Absorption maximum (λ_abs max_): 420–430 nm Emission (λ_em_): 650–680 nm Singlet oxygen quantum yield (Φ_Δ_): 0.5–0.6	Cell viability was significantly decreased in chlorophyll-PDT-treated cells, amplifying the anticancer efficacy against renal cell carcinoma cells	[102]
2025	Cancer photodynamic therapy enabled by water-soluble chlorophyll protein	Chlorophyll derivatives	PDT light dose: 660 nm, 10–20 J/cm^2^ Excitation (λ_ex_): 1530 nm Absorption (λ_abs_): 400–450 nm (Soret band), 660 nm (Q band) Absorption maximum (λ_abs max_): 660 nm Emission (λ_em_): 670–680 nm Singlet oxygen quantum yield (Φ_Δ_): 0.5–0.6	Water-soluble chlorophyll derivatives were tetrameric and stable under air/thermal conditions, and produced high ROS under red/far-red light irradiation to induce cancer cell death	[103]
2026	Antimicrobial photodynamic therapy using sodium iron chlorophyllin against drug-resistant *Cutibacterium acnes* from patients with acne vulgaris	Chlorophyll derivatives	PDT light dose: 660 nm, 15 J/cm^2^ Excitation (λ_ex_): 1530 nm Absorption (λ_abs_): 400–450 nm (Soret band), 620–660 nm (Q band) Absorption maximum (λ_abs max_): 660 nm Emission (λ_em_): 670–680 nm Singlet oxygen quantum yield (Φ_Δ_): 0.5–0.6	Chlorophyll-PDT was a promising nonantibiotic therapy for drug-resistant acne vulgaris, with potential implications for eradicating *C. acnes* colonization and preventing infections	[104]
2024	Enhancing the photosensitivity of hypocrellin A by perylene diimide metallacage-based host–guest complexation for photodynamic therapy	Hypocrellin A	PDT light dose: 630 nm, 10–15 J/cm^2^ Excitation (λ_ex_): 1530 nm Absorption (λ_abs_): 450–470 nm (blue), 620–640 nm (red) Absorption maximum (λ_abs max_): 630 nm Emission (λ_em_): 650–670 nm Singlet oxygen quantum yield (Φ_Δ_): 0.7–0.8	HA with perylene diimide metallacage-based host–guest complexation as an energy donor to promote the singlet oxygen generation ability via fluorescence resonance energy transfer, thereby achieving highly efficient PDT in cancer	[105]
2025	A glutathione responsive photosensitizer based on hypocrellin B for photodynamic therapy	Hypocrellin B	PDT light dose: 10–20 J/cm^2^ at 630 nm Excitation (λ_ex_): Multi-band 808 nm, 980 nm, 1530 nm Absorption (λ_abs_): 450–470 nm (blue), 620–640 nm (red) Absorption maximum (λ_abs max_): 630 nm Emission (λ_em_): 650–670 nm Singlet oxygen quantum yield (Φ_Δ_): 0.6–0.7	HB-NBD-PDT was activated by GSH in solutions and cancer cells, and obtained recuperative fluorescence and photosensitive activity for cancer cells	[106]
2025	Dual-responsive peptide-photosensitizer conjugate based on hypocrellin derivative for tumor-targeted photodynamic therapy	Hypocrellin derivatives	PDT light dose: 630 nm, 10–20 J/cm^2^ Excitation (λ_ex_): Multi-band 808 nm, 980 nm, 1530 nm Absorption (λ_abs_): 450–470 nm (blue), 620–640 nm (red) Absorption maximum (λ_abs max_): 630 nm Emission (λ_em_): 650–670 nm Singlet oxygen quantum yield (Φ_Δ_): 0.6–0.7	HB-HCPP-RGD-PDT possessed strong phototoxicity towards tumor cells, which actively targeted the tumor and achieved a high tumor inhibition rate with a high ROS yield	[107]
2025	Comparative study of free and encapsulated hypocrellin B on photophysical-chemical properties, cellular uptake, subcellular distribution, and phototoxicity	Hypocrellin B	PDT light dose: 630 nm, 10–20 J/cm^2^ Excitation (λ_ex_): Multi-band 808 nm, 980 nm, 1530 nm Absorption (λ_abs_): 450–470 nm (blue), 620–640 nm (red) Absorption maximum (λ_abs max_): 630 nm Emission (λ_em_): 650–670 nm Singlet oxygen quantum yield (Φ_Δ_): Free: 0.5–0.6, Encapsulated: 0.6–0.7	HB-PDT induced pronounced phototoxicity with substantial ROS production, which exhibited strong lysosomal colocalization and a unique intracellular trafficking pathway	[108]
2026	Caged-hypocrellin-mediated antimicrobial photodynamic therapy as a dual-action strategy for fungal clearance and immune response regulation in drug-resistant *Candida auris* wound infections	Hypocrellin	PDT light dose: 630 nm, 10–20 J/cm^2^ Excitation (λ_ex_): Multi-band 808 nm, 980 nm, 1530 nm Absorption (λ_abs_): 470–490 nm Absorption maximum (λ_abs max_): 480 nm Emission (λ_em_): 610–620 nm Singlet oxygen quantum yield (Φ_Δ_): 0.55–0.60	HB-PDT significantly reduced fungal burden and accelerated wound healing, which enhanced local infiltration of myeloid cells, B cells, γδ T cells, and type 2 innate lymphoid cells, while controlling systemic inflammation	[109]

**Table 6:** Natural product-based lanthanide complexes and UCNP systems in PDT.

Year	Topic	PS	Parameter(s)	Consequence	Reference
2017	Photophysics of coumarin- and carbostyril-sensitized luminescent lanthanide complexes: implications for complex design in multiplex detection	Coumarin and carbostyril ligands sensitizing Eu^3+^ and Tb^3+^ complexes	PDT light dose: not reported Excitation (λ_ex_): 980 nm Absorption (λ_abs_): UV–visible region (320–420 nm, coumarin ligand absorption) Absorption maximum (λ_abs max_): 350–370 nm depending on coumarin substitution Emission (λ_em_): Eu^3+^ red emission (615 nm), Tb^3+^ green emission (545 nm) Singlet oxygen quantum yield (Φ_Δ_): Φ_Δ_ not reported	Ln–coumarin complexes with antennae exhibit strong visible emission, high quantum yields, and efficient energy transfer, and photophysical properties suggest significant potential for the PDT application	[119]
2020	Coumarin–lanthanide-based compounds with SMM behavior and high quantum yield luminescence	Coumarin–lanthanide complexes (Eu^3+^/Tb^3+^/Dy^3+^/Tm^3+^)	PDT light dose: not reported Excitation (λ_ex_): 980 nm Absorption (λ_abs_): UV–visible region (350–450 nm, coumarin ligand absorption) Absorption maximum (λ_abs max_): 370–390 nm depending on coumarin substitution Emission (λ_em_): Tb^3+^ (green ~545 nm), Eu^3+^ (red ~615 nm), Dy^3+^ (yellow), Tm^3+^ (blue) Singlet oxygen quantum yield (Φ_Δ_): Φ_Δ_ not reported	Luminescent emission in the visible range was observed for all Ln–coumarin complexes upon ligand sensitization, which possessed high quantum yields to suggest strong potential for PDT application	[120]
2016	Riboflavin photoactivation by upconversion nanoparticles for cancer treatment	Riboflavin (NaYF_4_:Yb^3+^/Er^3+^)	PDT light dose: NIR excitation at 975 nm (continuous-wave diode laser), UCNPs convert NIR to visible light to activate riboflavin Excitation (λ_ex_): 980 nm Absorption (λ_abs_): Riboflavin absorbs in the blue–green region at 450 nm Absorption maximum (λ_abs max_): 445–450 nm (riboflavin core absorption) Emission (λ_em_): Riboflavin fluorescence at 520 nm, UCNP emission bands in green at 540 nm, and red at 650 nm used for riboflavin activation Singlet oxygen quantum yield (Φ_Δ_): 0.5	Riboflavin-UCNP energy transfer was photon-mediated with ~14% Förster process contribution, which was the NIR photodynamic treatment of the lesions	[121]
2025	Engineered riboflavin–cerium oxide nanoparticles for enhanced phototoxicity toward triple-negative breast cancer cells	Riboflavin conjugated with cerium oxide nanoparticles (CeO_2_ NPs)	PDT light dose: 450 nm, Blue light irradiation Excitation (λ_ex_): 980 nm Absorption (λ_abs_): Riboflavin absorbs in the blue–green region at 450 nm Absorption maximum (λ_abs max_): 445–450 nm (riboflavin core absorption) Emission (λ_em_): Riboflavin fluorescence at 520 nm Singlet oxygen quantum yield (Φ_Δ_): 0.5	Riboflavin–CeO_2_ NPs served as a selectively light-activated nanoplatform for PDT, which integrated redox functionality and photoactivity	[122]
2014	Upconversion nanoparticles conjugated with curcumin as a photosensitizer to inhibit methicillin-resistant *Staphylococcus aureus* in lung under near-infrared light	Curcumin conjugated to lanthanide-doped UCNPs (NaYF_4_:Yb^3+^/Er^3+^)	PDT light dose: 980 nm NIR irradiation, 10 J cm^−2^ Excitation (λ_ex_): 980 nm Absorption (λ_abs_): 980 nm (Yb^3+^ sensitization in UCNPs) Absorption maximum (λ_abs max_): 980 nm (strong UCNP absorption band) Emission (λ_em_): 540 nm (visible green, Er^3+^) and 650 nm (red) Singlet oxygen quantum yield (Φ_Δ_): 0.4–0.5 nm	UCNPs-curcumin was developed, which produced singlet oxygen and reached a stable level after 30 min of irradiation, for the effect on MRSA through bacterial cytoplasm leakage	[123]
2020	Photophysical, theoretical and photo-cytotoxic evaluation of a new class of lanthanide(III)–curcumin/diketone complexes for PDT application	Curcumin acts as β-diketonate ligand and fluorophore	PDT light dose: Visible light from 400 to 700 nm, 10 J cm^−2^ Excitation (λ_ex_): 980 nm Absorption (λ_abs_): 400–700 nm (broad visible region) Absorption maximum (λ_abs max_): 430–450 nm Emission (λ_em_): 520–550 nm Singlet oxygen quantum yield (Φ_Δ_): >0.6	Lanthanide(III)–curcumin/diketonecomplexes facilitated ISC in curcumin by reducing the energy gap of the singlet-to-triplet excited state, increasing the generation of singlet oxygen with the photo-cytotoxicity in HeLa and MCF-7 cells	[124]
2021	Nanocurcumin-loaded UCNPs for cancer theranostics: physicochemical properties, in vitro toxicity, and in vivo imaging studies	Curcumin lanthanide-doped UCNPs (NaYF_4_:Yb^3+^/Er^3+^)	PDT light dose: 980 nm NIR irradiation, 10 J cm^−2^ Excitation (λ_ex_): 980 nm Absorption (λ_abs_): 980 nm (Yb^3+^ sensitization in UCNPs) Absorption maximum (λ_abs max_): 980 nm (strong UCNP absorption band) Emission (λ_em_): 540 nm (visible green, Er^3+^) and 650 nm (red) Singlet oxygen quantum yield (Φ_Δ_): 0.4–0.5	Curcumin-loaded nanocomplexes were developed in tumor-bearing laboratory animals (Lewis lung cancer model), which indicated adequate contrast to enable in vivo and ex vivo study of UCNP-PLGA-nanocurcumin biodistribution in organs, with dominant distribution in the liver and lungs	[125]
2022	Construction and evaluation of curcumin upconversion nanocarriers decorated with MnO2 for tumor photodynamic therapy	Curcumin loaded on lanthanide-doped UCNPs (NaYF_4_:Yb^3+^/Er^3+^) with MnO_2_ shell	PDT light dose: 980 nm NIR irradiation, 10 J cm^−2^ Excitation (λ_ex_): 980 nm Absorption (λ_abs_): 980 nm (Yb^3+^ sensitization in UCNPs) Absorption maximum (λ_abs max_): 980 nm (strong UCNP absorption) Emission (λ_em_): 540 nm (visible green, Er^3+^) Singlet oxygen quantum yield (Φ_Δ_): > 0.5	Curcumin played a dual role not only as a PS, but also as a chemotherapeutic agent, which existed with MnO2-decorated upconversion nanoparticles to solve the tissue penetration and tumor hypoxic microenvironment for tumor PDT	[126]
2024	Near-infrared light-activatable upconversion nanoparticle/curcumin hybrid nano-drug: a potent strategy to induce the differentiation and elimination of glioma stem cells	Curcumin conjugated to lanthanide-doped UCNPs (NaYF_4_:Yb^3+^/Er^3+^)	PDT light dose: 980 nm NIR irradiation, 10 J cm^−2^ Excitation (λ_ex_): 808 nm Absorption (λ_abs_): 980 nm (Yb^3+^ sensitization in UCNPs) Absorption maximum (λ_abs max_): 980 nm (strong UCNP absorption band) Emission (λ_em_): 540 nm (visible green, Er^3+^) and 650 nm (red) Singlet oxygen quantum yield (Φ_Δ_): 0.45–0.55	UCNPs-F127@Cur-PDT caused cell cycle arrest and induced the differentiation of glioma stem cells by suppressing the Wnt-β-catenin and Jak-Stat signaling pathways	[127]
2026	A MOF-lanthanide theranostic agent with bidirectional near-infrared photon conversion for tumor-responsive therapy and real-time imaging	MOF-lanthanide agent with curcumin chromophores	PDT light dose: 808 nm or 980 nm NIR irradiation, 10 J cm^−2^ Excitation (λ_ex_): 808 nm Absorption (λ_abs_): 808–980 nm NIR region Absorption maximum (λ_abs max_): 980 nm (Yb^3+^ sensitization) Emission (λ_em_): 540 nm (Er^3+^, green), 1530 nm (NIR-II, Er^3+^) Singlet oxygen quantum yield (Φ_Δ_): > 0.6	A MOF-lanthanide theranostic agent achieved a synergistic chemotherapy/CDT/PDT effect, with about 80% cell-killing efficiency and a 15-fold tumor inhibition, which was an efficient theragnostic agent for precision oncology	[128]
2012	Pyropheophorbide a and c(RGDyK) co-modified chitosan-wrapped upconversion nanoparticle for targeted near-infrared photodynamic therapy	Chlorophyll derivatives, pyropheophorbide a (PPa) conjugated to lanthanide-doped UCNPs (NaYF_4_: Yb^3+^/Er^3+^), surface wrapped with chitosan and c(RGDyK) peptide	PDT light dose: 980 nm NIR irradiation, 10 J cm^−2^ Excitation (λ_ex_): 808 nm Absorption (λ_abs_): 980 nm (Yb^3+^ sensitization in UCNPs) Absorption maximum (λ_abs max_): 980 nm (UCNP core absorption); 670 nm Pyro-A absorbs in visible red Emission (λ_em_): 540 nm (visible green, Er^3+^), 650 nm (red, Er^3+^), red emission overlaps 670 nm Pyro-A absorption Singlet oxygen quantum yield (Φ_Δ_): 0.52–0.54	UCNP-PPa-RGD was developed, which demonstrated a highly stable and efficient PS-modified upconversion nanostructure for targeted NIR PDT of cancer cells.	[129]
2020	Near-infrared light-initiated upconversion nanoplatform with tumor microenvironment responsiveness for improved photodynamic therapy	Chlorophyll derivatives, pyropheophorbide-a (PPa) loaded into the mesoporous silica shell of UCNPs, NaGdF_4_:Yb^3+^/Er^3+^, NaGdF_4_:Yb^3+^/Nd^3+^	PDT light dose: 808 nm NIR irradiation, 10 J cm^−2^ Excitation (λ_ex_): 808 nm Absorption (λ_abs_): 808 nm (Nd^3+^ sensitization in UCNPs) Absorption maximum (λ_abs max_): 808 nm (Nd^3+^ band); PPa absorbs in visible green at 540 nm and red from 650 to 670 nm Emission (λ_em_): 540 nm (green, Er^3+^) and 650 nm (red, Er^3+^) Singlet oxygen quantum yield (Φ_Δ_): 0.79	UCNP@SiO2/PPa&DOX@Cs-FA nanoplatform was responsive to the tumor microenvironment, integrating low-intensity light excitation, slow release, targeting, PDT, and chemotherapy	[130]
2003	The photodynamic property improvement of hypocrellin A by chelation with lanthanum ions	Hypocrellin A chelated with lanthanum ions (La^3+^–HA complex)	PDT light dose: not reported Excitation (λ_ex_): 1530 nm Absorption (λ_abs_): Broad absorbance in the phototherapeutic window from 600 to 800 nm Absorption maximum (λ_abs max_): not reported Emission (λ_em_): Not reported Singlet oxygen quantum yield (Φ_Δ_): not reported	Lanthanum ion with hypocrellin A (La^3+^-HA) possessed high singlet oxygen generation efficiency, large absorbance in the phototherapeutic window, and great water solubility	[131]
2008	Photodynamic potentiality of hypocrellin B and its lanthanide complexes	Hypocrellin B –lanthanide complexes (Eu^3+^/La^3+^/Tb^3+^)	PDT light dose: White light exposure (photobleaching observed after 30 min) Excitation (λ_ex_): 1530 nm Absorption (λ_abs_): blue–green visible region Absorption maximum (λ_abs max_): Red-shifted by 30 nm in HB–La^3+^ complex Emission (λ_em_): HB fluorescence in red from 610 to 620 nm Singlet oxygen quantum yield (Φ_Δ_): 0.32	HB–lanthanide complexes induced the larger red shift of about 30 nm, as well as enhanced singlet oxygen generation quantum yield in PDT	[132]
2013	A new near-infrared photosensitizing nanoplatform containing blue-emitting upconversion nanoparticles and hypocrellin A for photodynamic therapy of cancer cells	Hypocrellin A loaded on blue-emitting UCNPs, NaYbF_4_, NaGdF_4_:Tm^3+^/Gd^3+^	PDT light dose: 980 nm NIR irradiation (upconversion to blue emission) Excitation (λ_ex_): 1530 nm Absorption (λ_abs_): HA absorbs strongly in the blue region from 470 to 480 nm Absorption maximum (λ_abs max_): 475 nm (blue emission from UCNPs overlaps with HA absorption) Emission (λ_em_): UCNP emission peaks in blue at 475 nm, HA fluorescence in red from 610 to 620 nm Singlet oxygen quantum yield (Φ_Δ_): 0.62	UCNPs@HA complexes possessed strong blue upconversion luminescence and good water dispersibility, as well as efficiently produced singlet oxygen to kill cancer cells in PDT	[133]
2022	Orthogonal excitations of lanthanide nanoparticle up/downconversion emissions via switching NIR lights for in vivo theranostics	Hypocrellin A loaded into the mesoporous silica shell of UCNPs, NaGdF_4_:Yb^3+^/Er^3+^, NaGdF_4_:Yb^3+^/Nd^3+^	PDT light dose: 808 nm and 980 nm NIR irradiation (orthogonal switching), 10–15 J cm^−2^ Excitation (λ_ex_): 1530 nm Absorption (λ_abs_): 808 nm (Nd^3+^ sensitization), 980 nm (Yb^3+^ sensitization) Absorption maximum (λ_abs max_): 808 nm and 980 nm (UCNP core); HA absorbs in visible red from 610 to 620 nm Emission (λ_em_): 540 nm (green, Er^3+^) and 650 nm (red, Er^3+^) Singlet oxygen quantum yield (Φ_Δ_): 0.79	HA-CSNP was developed and modified with Cy-GSH to quench NIR-IIb emission, which demonstrated a high tumor-to-normal tissue ratio in vivo	[134]
2025	Rational designing of hypocrellin B-loaded mesoporous silica-NaYF_4_@NaSmF_4_ Core@Shell upconversion nanoparticles for targeted in vitro breast cancer therapy	Hypocrellin B loaded in the mesoporous silica shell of NaYF_4_@NaSmF_4_ UCNPs	PDT light dose: 980 nm NIR excitation Excitation (λ_ex_): Multi-band 808 nm, 980 nm, 1530 nm Absorption (λ_abs_): HB absorbs in the blue–green visible region from 470 to 500 nm Absorption maximum (λ_abs max_): 480 nm (UCNP emission band) Emission (λ_em_): UCNP emission overlaps HB absorption, HB fluorescence in red from 610 to 620 nm Singlet oxygen quantum yield (Φ_Δ_): 0.6	HB-mSi-CS-UCNPs represent higher singlet oxygen production, leading to cancer cell death by either apoptosis or necrosis for the treatment of breast cancers	[135]

**Table 7:** Comparison of conventional use and lanthanide-enhanced systems.

Feature(s)	Conventional Natural PSs	Lanthanide-Enhanced Systems
**Excitation**	UV–visible light from 350 to 450 nm	808–980 nm NIR
**Penetration**	Shallow	Deep
**Photostability**	Photobleaching and degradation	More stable
**Generation of ROS**	0.2–0.6	>0.6
**Selectivity**	Effective but sometimes non-specific	Depend on the design of UCNP
**Limitations**	Shallow penetration, low quantum yield, instability	More complex synthesis

## Data Availability

No new data were created or analyzed in this study. Data sharing is not applicable.

## Abbreviations

The following abbreviations are used in this manuscript:

ALA	5-aminolevulinic acid
Ce6	Chlorin e6
CM@UCNP-RB/PDT	Cancer-cell-biomimetic UCNP with rose bengal/photodynamic therapy
FRET	Förster resonance energy transfer
FUCP-1	Fluorescent upconversion photosensitizer 1
Gd_2_O_3_	Gadolinium oxide
ISC	Intersystem crossing
MRI	Magnetic resonance imaging
MOF	Metal–organic framework
NIR	Near-infrared
NIR-I	Near-infrared window I (700–950 nm)
NIR-II	Near-infrared window II (1000–1700 nm)
PDT	Photodynamic therapy
Φ_Δ_	Singlet oxygen quantum yield
PS	Photosensitizer
PTT	Photothermal therapy
REEs	Rare-earth elements
RB	Rose bengal
ROS	Reactive oxygen species
TPP	Triphenylphosphine
UCNPs	Upconversion nanoparticles
UDNPs	Upconversion–downshifting nanoparticles
UV-vis	Ultraviolet–visible
ZnPc	Zinc phthalocyanine
